# Recent advances in carbon-based materials for high-performance perovskite solar cells: gaps, challenges and fulfillment

**DOI:** 10.1039/d3na00005b

**Published:** 2023-02-17

**Authors:** Sandeep Pandey, Manoj Karakoti, Dinesh Bhardwaj, Gaurav Tatrari, Richa Sharma, Lata Pandey, Man-Jong Lee, Nanda Gopal Sahoo

**Affiliations:** a Department of Chemistry, Konkuk University Seoul 05029 Republic of Korea leemtx@konkuk.ac.kr; b Liquid Crystals Research Center, Konkuk University Seoul 05029 Republic of Korea; c PRS Nanoscience and Nanotechnology Centre, Department of Chemistry, Kumaun University D.S.B. Campus Nainital-263001 Uttarakhand India ngsahoo@yahoo.co.in; d Vikas Ecotech Limited 34/1 East Punjabi Bagh New Delhi-110026 India; e Chemistry of Interface, Lulea Technology University Lulea Sweden; f Maharaja Agrasen Institute of Technology GGSIPU, Rohini New Delhi 110086 India; g Research Institute for Green Energy Convergence Technology, Gyeongsang National University Jinju 52828 Republic of Korea

## Abstract

Presently, carbon-based nanomaterials have shown tremendous potential for energy conversion applications. Especially, carbon-based materials have emerged as excellent candidates for the fabrication of halide perovskite-based solar cells, which may lead to their commercialization. In the last decade, PSCs have rapidly developed, and these hybrid devices demonstrate a comparable performance to silicon-based solar cells in terms of power conversion efficiency (PCE). However, PSCs lag behind silicon-based solar cells due to their poor stability and durability. Generally, noble metals such gold and silver are employed as back electrode materials during the fabrication of PSCs. However, the use of these expensive rare metals is associated with some issues, urgently necessitating the search for cost-effective materials, which can realize the commercial applications of PSCs due to their interesting properties. Thus, the present review shows how carbon-based materials can become the main candidates for the development of highly efficient and stable PSCs. Carbon-based materials such as carbon black, graphite, graphene nanosheets (2D/3D), carbon nanotubes (CNTs), carbon dots, graphene quantum dots (GQDs) and carbon nanosheets show potential for the laboratory and large-scale fabrication of solar cells and modules. Carbon-based PSCs can achieve efficient and long-term stability for both rigid and flexible substrates because of their high conductivity and excellent hydrophobicity, thus showing good results in comparison to metal electrode-based PSCs. Thus, the present review also demonstrates and discusses the latest state-of-the-art and recent advances for carbon-based PSCs. Furthermore, we present perspectives on the cost-effective synthesis of carbon-based materials for the broader view of the future sustainability of carbon-based PSCs.

## Abstract 2

Recent advances on the carbon-based materials for efficient and stable perovskite solar cells (PSCs) is deeply analysed. Present review discussed about gaps, challenges, and potential fulfilment towards the possible commercialization of carbon-PSCs.

## Graphical abstract

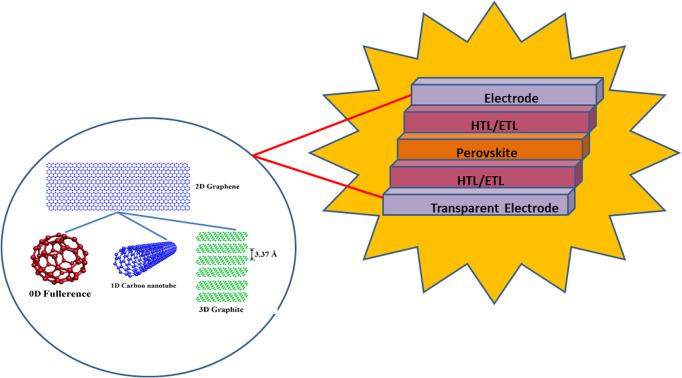

## 1. Introduction

Carbon-based materials are positioned among the top materials existing on Earth, where carbon is one of the most abundant elements. The availability of a variety of amazing allotropes of carbon makes it very popular for various types of applications including energy harvesting applications.^1^ Among the various forms of carbon, 1D, 2D, and 3D carbon nanomaterials have gained special attention for energy conversion applications because of their extraordinary mechanical, electrical, optical, and chemical properties.^2^ Activated carbon, carbon black and graphite show unique three-dimensional structural properties, making them suitable for a variety of energy harvesting applications.^3–6^ Alternatively, carbon nanomaterials such as graphene, carbon nanotubes (CNTs), graphene quantum dots (GQDs) and carbon quantum dots (CQDs) demonstrate unique properties for a variety of energy conversion applications.^7–12^ The extraordinary properties such as electrical, mechanical, and optical properties of these carbon-based materials present excellent opportunities to develop diverse carbon-based photovoltaic applications. Especially, these carbon-based materials showed promising behavior for the fabrication of perovskite solar cells (PSCs), where their stability and power conversion efficiency (PCE) will decide the future of their commercialization.^13^ PSCs have attracted significant interest because of the potential to engineer large-scale semi-transparent and transparent flexible devices.^14,15^ Currently, the state-of-the-art of perovskite-based photovoltaic devices are based on organometallic compounds, *i.e.*, organotin or organolead halide perovskite, as the active layer for light-harvesting in PSCs. Generally, perovskites have the formula of ABX_3_, which are labelled specifically as methyl ammonium (MA) lead (Pb) trihalide (X), *i.e.*, MAPbX_3_ or CH_3_NH_3_PbX_3_, where X is a halogen ion (Cl^−^, Br^−^, and I^−^). These lead- or tin-based organometal halides are generally used as the light-absorbing layer in PSCs, which possess several advantages such as direct bandgap, solution processability, high light absorption coefficient and long electron–hole diffusion length (approx. 100 nm for CH_3_NH_3_PbI and approx. 1 mm for CH_3_NH_3_PbI_3−*x*_Cl_*x*_) and high carrier mobility. The simplified design of PSCs includes three main layers including a hole transport layer (p-type semiconductor), light-absorbing active perovskite layer (MAPBX_3_), and electron transport layer (n-type semiconductor). Thus, when the light-absorbing layer absorbs visible light, it generates a negatively charged electron and positively charge hole, which are subsequently transported towards the opposite electrodes by the HTL and ETL layers to form a loop.^16–21^ The main factors that affect the performance of PSCs include the film morphology, surface and interface uniformity, thickness, material composition, deposition method and mechanism of the designed materials. PSCs are very sensitive to moisture, and thus long-term stability is one of the critical parameters, which is still required to be controlled by surface chemistry to avoid indirect atmospheric effects on PCEs and stability of PSCs.^22^ Initially, perovskite materials were used in DSSCs, where the first report on perovskite-DSSCs was presented in a conference paper. This paper showed the successive deposition of MAPbI_3_ nanocrystals over a layer of TiO_2_ having a thickness of 8 μm. A PCE of 3.81% was achieved for the perovskite-based DSSC.^23^ Subsequently, another perovskite-based DSSC was reported with an improved efficiency of 6.54% by modifying the electrolyte system.^24^

However, although research has shown that organic–inorganic perovskite materials can be successfully employed to replace the conventional molecular dyes in DSSCs, the instability issues of perovskite materials in the presence of polar electrolyte systems hinders the development of perovskite-based DSSCs. Because of this, no significant research was reported from 2009 to 2011. However, a breakthrough was obtained in the development of PSCs in 2012, when the first solid-state perovskite solar cell was demonstrated with an improved efficiency of 9.7% and long-term stability of 500 h without any encapsulation by replacing the liquid electrolyte with the organic hole conductor of 2,2′7,7′-tetrakis-(*N*,*N*-di-*p*-methoxyphenyl amine)-9,9′-spirobifluorene (spiro-MeOTAD).^25^ After this breakthrough, research on PSCs has rapidly increased to enhance their stability and PCEs. Several efforts have been devoted to the different layers of PSCs to improve the device parameters. Some of the modifications include improvement of the conductivity of the electrodes, enhancement in the charge extraction capability of the ETL and HTL, modifying the absorbance efficiency of the perovskite layer by doping and developing carbon-based PSCs.^26–29^ In the last few years, PSCs have achieved a record efficiency of 25.7%.^30,31^ However, despite this, these solar cells still lag behind the first-generation and second-generation solar cells because of their stability issues. In this case, carbon-based materials have been shown to have extensive advantages for the fabrication of PSCs due to their wide scalability in almost every part of PSCs. These materials can be used as the HTL and ETL for better charge extraction or as TCEs based on their optical properties. The major applicability of carbon-based materials is considered as the back electrode material in carbon-based PSCs. Due to the hydrophobic property and enhanced conductivity of carbon-based materials, both the stability and PCEs of PSCs can be improved, as suggested by previous reports.^32^ Presently, researchers are focusing on large-area PSC modules by utilizing the properties of carbon-based materials. Especially, efforts have been devoted to searching for efficient carbon-based materials that not only provide stability to PSCs, but also improve their PCEs. Additionally, the cost of the material should be suitable for its use in a commercial platform, where a wide range of users can benefit from low-cost carbon for the development of cost-effective PSCs. Hence, several new routes for the synthesis of carbon-based materials have also been explored in recent years. Techniques for the mass-scale production of graphene, CNTs, carbon spheres, carbon nanosheets, graphene quantum dots (GQDs), carbon quantum dots (CQDs), graphene nanofibers (GNFs) and several other forms of carbon with low cost have also been demonstrated in the last few years,^33–36^ indicating that low-cost carbon is also available for the wide scalability of carbon-based PSCs. Thus, the present review is focused on the recent progress on carbon-based PSCs, where carbon-based materials were mainly used as the back electrode material in efficient and stable PSCs. Further, we also highlight the recently developed methods for the synthesis of carbon nanomaterials and carbon-based nanocomposites from different precursor materials. The optimization and modification of these methods revealed an opportunity for the mass-scale production of carbon-based materials for energy harvesting applications. Further, our analysis from and extensive literature survey showed that conducting forms of carbon can be used as versatile materials in different parts of PSCs, and thus show the future sustainability of carbon-based materials for the large-scale development of PSCs. In this review, we also present an overview of the various strategies to improve the PCEs and stability of PSCs *via* functionalization and engineering methods. Furthermore, we demonstrate the application of carbon based TCEs for PSCs and their sustainability in large-scale PSCs. Finally, we present the best options for the circular economy analysis together with cost-benefit investigation of cost-effective solutions for the large-scale production of carbon-based PSCs.

## 2. Superiority of carbon-based materials compared to other materials

Today, carbon-based materials are extensively used as electrode materials, charge transporting materials and doping materials in the active layer of PSCs. Because of the excellent hydrophobic property of carbon-based materials, PSCs with carbon-based electrodes show excellent stability in comparison to that with metal-based electrode materials. Generally, perovskite degradation is initiated in the presence of moisture, which occurs because of the formation of hydrated phases of perovskites such as CH_3_NH_3_PbI_3_·H_2_O and (CH_3_NH_3_)_4_PbI_6_·2H_2_O.^37^ Thus, PSCs need to be protected from moisture, but can be avoided by using carbon-based electrodes. Further, the use of carbon-based electrodes in PSCs also presents the possibility of cost-effective perovskite solar modules, and thus the development of carbon-based PSCs has attracted interest from researchers. Graphite, 2D/3D graphene nanosheets, carbon black, GQDs and CQDs have demonstrated utility as efficient electrode materials.^32^ However, a huge debate is regarding the superiority and selectivity of the potential form of carbon for the development of carbon electrodes in PSCs. Specifically, the development of carbon-based electrodes for PSCs is hindered by the poor dispersion properties of carbon-based materials in a variety of solvent systems. Therefore, equipment such as ultrasonic homogenizers and high energy ball mills is required for the proper dispersion of carbon-based materials. Alternatively, the growth of carbon-based nanomaterials for electrode applications shows their applicability for the large-scale production of PSCs, but the use of expensive substrates and sensitive instrumentation handling also limit their wide-scale applicability. Recently, efforts have been devoted to the cost-effective synthesis of carbon-based nanomaterials and their applications for energy harvesting applications. In this regard, several new routes for the synthesis of carbon nanomaterials from carbon-containing precursors have been identified.^33^ In the next section, we discuss the different routes for the synthesis of carbon nanomaterials using carbon-containing precursors.

## 3. Methods for the synthesis of carbon nanomaterials

### 3.1 Synthesis routes for graphene

**Fig. 1:**
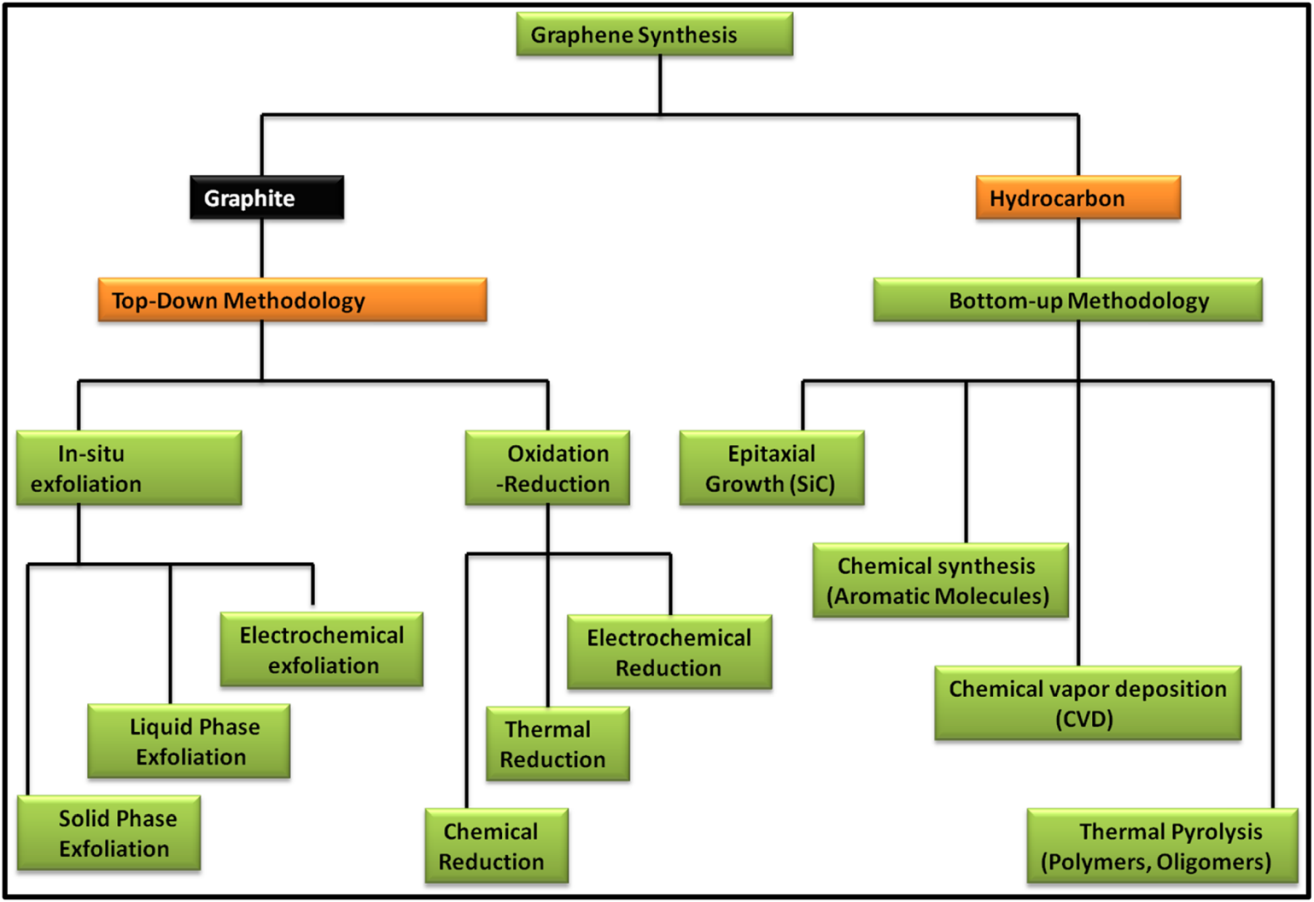
Schematic representation of the methods for the synthesis of graphene *via* top-down and bottom-up approaches.

Since the discovery of carbon nanomaterials, various routes for their synthesis have been explored. In this case, two main carbon nanomaterials, *i.e.*, CNTs and graphene, have been widely explored in recent years. Especially, graphene has attracted tremendous interest from researchers because of its exceptional properties such as high surface area and extraordinary optical, electrical and mechanical properties. Since the discovery of graphene in 2004, numerous reports and patents have been published, which is continuously increasing daily.^38^ In recent years, several review articles showed the continuous progress of the applications of graphene and graphene-based nanocomposite materials. Specifically, they showed the latest outcomes of techniques for the production of the graphene and their associated environmental impact. In this regard, various methods have been discussed and reviewed, including chemical vapour deposition (CVD), mechanical exfoliation and chemical exfoliation.^39–45^ In addition to the synthesis methods, characterization techniques such Raman spectroscopy were reviewed by Wu *et al.*, where they showed the different aspects of Raman spectroscopy based on fundamental and practical research on graphene and graphene-related applications.^46^ Further, Phiri *et al.* revealed the various routes for the synthesis of these materials in their review article, which focused on the synthesis of graphene from graphite and discussed the application of graphene in polymer nanocomposites.^47^ Another study focused on the routes available for the synthesis of nanoporous graphene-based materials for various applications.^48^ Several methods are available for the synthesis of graphene, which can be classed into two main approaches, *i.e.*, top-down and bottom-up approaches. These routes focus on quality control assessment, which raises the question of the mass-scalability of the production techniques, while maintaining their cost benefit. Fig. 1 shows the various routes for the synthesis of graphene based on the top-down and bottom-up approaches.

In the top-down approach for the synthesis of graphene, graphite is employed as the precursor, which is majorly done *via in situ* exfoliation and oxidation–reduction methods. The *in situ* exfoliation method for the synthesis of graphene involves: (1) solid-phase exfoliation by micromechanical and ball milling techniques, (2) liquid-phase exfoliation, including exfoliation by sonication, wet ball milling and shearing in various types of solvent systems and (3) electrochemical exfoliation. Meanwhile, the oxidation–reduction method involves: (1) chemical reduction using general reducing agents such as hydrazine, metal hydrides, amines, ammonia and alcohols and (2) thermal reduction *via* several techniques such as hydrothermal, solvothermal, photothermal and thermal annealing and electrochemical reduction. Alternatively, in the bottom-up methodology, hydrocarbon is employed as the precursor material for the synthesis of graphene together with various approaches such as epitaxial growth using SiC, chemical synthesis using aromatic molecules, CVD using common hydrocarbons such as CH_4_, C_2_H_6_ and C_3_H_8_ and various forms of transition metals such as Cu and Ni and thermal pyrolysis of polymers and oligomers.

Recently, Ding *et al.* showed an eco-friendly green method for the mass-scale synthesis of few-layered graphene nanoplatelets from graphite in pure water with a thickness of 2.24, 0.52 and 1.76 nm, as confirmed by AFM analysis. This group introduced a facile liquid exfoliation route assisted by vapour pre-treatment for the synthesis of graphene nanoplatelets, while after the synthesis, transparent conducting films were fabricated. This method was demonstrated to be an eco-friendly way for producing cost-effective graphene-based materials for real-time applications,^49^ while Chen *et al.* reported the synthesis of few-layer graphene using graphite as the precursor material *via* physical sonication. This study used a chemically modified degradable water-soluble polymer and showed a production capacity of 6 g h^−1^.^50^ To demonstrate the utility of liquid-phase exfoliation assisted with sonication, Buzaglo *et al.* showed a continuous and semi-industrial sonication procedure in aqueous media to produce graphene sheets from graphite. They showed that a certain specific energy value was needed to exfoliate graphite to get high-quality graphene sheets, thereby validating the sonication method for the rapid production of graphene sheets.^51^

Another route for the synthesis of graphene is the utility of electrochemical exfoliation by using graphite, graphite foil or highly oriented pyrolytic graphite (HOPG) rods. Generally, aqueous or non-aqueous electrolyte solutions are employed in electrochemical exfoliation, thereby showing a cost-effective route for the synthesis of graphene sheets. In this regard, Coroş *et al.* reported a simple and cost-effective electrochemical exfoliation method with varying electrochemical parameters to produce high-quality graphene sheets in acidic electrolyte.^52^ The scanning electron microscopy (SEM) images of the as-produced graphene sheets showed a randomly arranged crumpled morphology, thereby indicating the overlapping of the graphene sheets. Munuera *et al.* reported the synthesis of low oxygen-content graphene sheets from graphite using sodium halide as the electrolyte, which was later investigated for dye adsorbents and electrodes for supercapacitors,^53^ while Hossain *et al.* used (NH_4_)_2_SO_4_ solution to obtain single-to double-layer graphene sheets from graphite rods in the temperature range of 25 °C to 90 °C without the use of H_2_O_2_.^54^ In addition to these electrolytes, several other electrolytes have also been reported to produce high-quality graphene sheets.

Shahriary *et al.* described a different method to synthesize graphene *via* the chemical technique, in which graphene oxide (GO) was firstly formed by using a strong oxidizing method, and then graphene oxide was reduced using a reducing agent or thermal reduction technique. The chemical exfoliation technique involves the oxidation of graphene *via* a modified Hummer's method,^55^ which involves the use of KMnO_4_ and H_2_SO_4_ to oxidize the graphitic skeleton. Subsequently, the obtained graphene oxide (GO) is reduced with the help of reducing agents such as hydrazine and sodium borohydride. Because of the toxic nature of these reducing agents, green reducing agents are highly desirable, and therefore used in recent years, as reported in the literature.^56–58^ Some of the green reducing agents reported in previous years include uric acid,^59^ ascorbic acid,^60^ tea leaf extract,^61^*Annona squamosa* leaf extract,^62^*Melissa officinalis* extract,^63^*Lycium barbarum* extract^64^ and caffeic acid.^65^ Thus, all the routes in the category of the top-down approach show a promising way for the production of high-quality graphene sheets.

Besides the top-down approach, which requires graphitic material for the synthesis of graphene sheets, several other methods have been explored by researchers, including the use of carbon-containing precursor molecules, rather than graphitic materials. These carbon-containing precursor molecules are converted to graphene sheets by different synthetic routes, which fall under the scope of the bottom-up approach. The epitaxial method, chemical synthesis, CVD and thermal pyrolysis are some of the well-known bottom-up approaches, which are popularly used for the synthesis of graphene sheets. The epitaxial method has been used to synthesize high-quality graphene sheets *via* the thermal decomposition of silicon carbide (SiC). The as-obtained epitaxial graphene can be used for electrical devices. In this regard, the synthesis of the non-exfoliated monolayer and single-layer graphene was demonstrated by Qin *et al.* on 4H-SiC substrates,^66^ while Mitsuhashi *et al.* showed the synthesis of uniform epitaxial graphene using 6H-SiC substrates at the annealing temperature of 1400–1900 °C.^67^ Yu *et al.* showed a new approach for the synthesis of high-quality transfer-free graphene by using cemented carbides and showed the importance of the epitaxial method for industrial applications.^68^ Among the bottom-up approaches, the CVD technique is regarded as one of the most popular techniques for the synthesis of high-quality graphene sheets. However, the CVD technique requires sophisticated instruments to control the synthesis parameters such as temperature, pressure, deposition, time, precursors, and type of catalytic system. Table 1 shows some of the previously reported work on the CVD method to synthesize various types of graphene.

**Table 1:** CVD methods for the synthesis of various types of graphene reported in the literature

CVD method	Substrate/precursor molecules	Temperature (°C)	Quality of graphene	Ref.
CVD	Cu/CH_4_	1000	Single-layer graphene	69
CVD	Cu/CH_4_	1030	High-quality graphene films with single crystalline properties	70
CVD	Cu/CH_4_	1060	Polycrystalline monolayer graphene	71
CVD	Cu/(H_2_ + CH_4_)	1070	Graphene single crystal	72
CVD with induction of heating	(AuCu + MgO or AgCu + MgO)/CH_4_	1000	Bimetallic nanoparticle-doped high-quality graphene sheets	73
CVD	PET and glass/10 nm thick Ti layers	150	Defect-free graphene	74
Plasma enhanced CVD	1,2-Dichlorobenzene/CH_4_	Without any active heating	Graphene nanostrips	75

Additionally, chemical synthesis using aromatic molecules has been shown as another route for the synthesis of graphene sheets. Moreno *et al.* reported the synthesis of porous graphene nanoribbons from aromatic dihalide monomers *via* a surface-assisted Ullmann coupling reaction. Firstly, they converted aromatic dihalide monomers into polymer chains, followed by cyclodehydrogenative aromatization to obtain graphene nanoribbons. Finally, the dehydrogenative cross-coupling of the graphene nanoribbons led to the formation of nanoporous graphene.^76^ Souza *et al.* showed the one-pot synthesis of graphene/polyaniline nanocomposites using benzene and aniline as precursor molecules. After the synthesis of the graphene/polyaniline nanocomposites, they were used as active layer materials for the application of supercapacitors.^77^ Further, alkyne benzannulation using Brønsted acid molecules was shown an another route for the synthesis of graphene *via* the bottom-up approach.^78^ Thus, it can be seen from the above-discussed literature review on the synthesis of graphene, both the top-down and bottom-up approaches have been used in the field of graphene synthesis. Briefly, inspection of these methods revealed that cost-effective and eco-friendly processes are still needed for the mass-scale production of graphene sheets, considering both industrial symbiosis and the circular economy.

### 3.2 Synthesis routes for CNTs

**Fig. 2:**
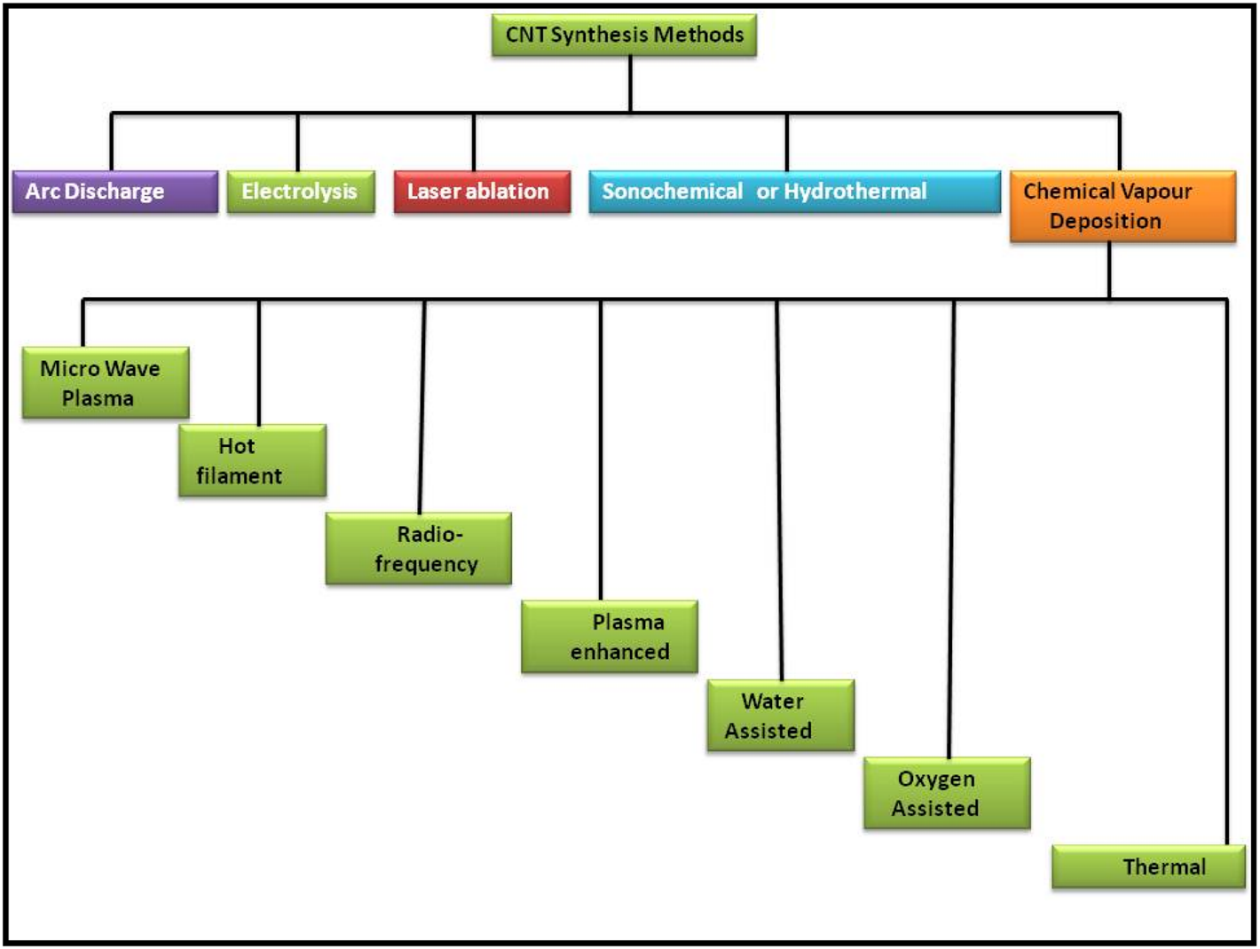
Schematic representation of some of the most applicable routes for the synthesis of CNTs.

CNTs as another allotrope of carbon are regarded as exceptional carbon nanomaterials because of their unique properties for various daily applications. Thus, the scientific community has been continuously researching CNTs from more than 20 years to determine the best route for their synthesis and fulfil the demand for cost-effective eco-friendly techniques. After the random discovery of CNTs in 1991 by Iijima while attempting to synthesize the C_60_ carbon molecule by the arc evaporation method, this new carbon allotrope gained significant attention.^79^ In continuation of their work, in 1993, Iijima *et al.* and Bethune *et al.* reported the synthesis of SWCNTs,^80,81^ which are regarded as the rolled form of single-layer graphite, *i.e.*, graphene. Because of the high electrical conductivity and promising optical transparency of CNTs, they can be employed in a wide variety of applications for the development of solar cells and other energy harvesting devices. Thus, a variety of routes has been proposed for the synthesis of different types of CNTs. Fig. 2 shows a schematic representation of some of the most applicable routes for the synthesis of CNTs including arc discharge, electrolysis process, laser ablation, sonication, hydrothermal method and CVD. Among them, the CVD route has been widely explored in the past few years. Besides this synthesis process, several new techniques such as microwave plasma, hot filament, radiofrequency, plasma-enhanced, water, oxygen and thermal treatment have also been used for the synthesis of CNTs.

Among these methods, the arc discharge method is one of the oldest methods to synthesize CNTs with fewer structural defects at a higher temperature of usually >1700 °C. Wang *et al.* reported the synthesis of CNTs *via* the DC arc discharge of graphite electrodes in He and methane. Thin and long MWCNTs were produced under the low gas pressure of 50 Torr and arc current of 20 A for the anode.^82^ Shimotani *et al.* used an He atmosphere and three organic solvents, *i.e.*, ethanol, alcohol and hexane, to increase the yield of MWCNTs in the arc discharge method.^83^ Jung *et al.* reported the large-scale synthesis of MWCNTs in a liquid nitrogen environment, and thereby showed that the arc discharge method can become a viable option for the mass production of MWCNTs. Further, it was observed that MWCNTs were formed in the absence of a catalyst, while SWCNTs were produced when transition metals were used as the catalyst. Usually, the synthesis of SWCNTs involves the use of an anode made up of graphite–metal composites, such as Fe, Ni, Co, Ag, Pd, and Pt or a combination of two metals such as Fe–Ni, Ni–Cu, Co–Ni, and Co–Cu. In this regard, Bethune *et al.* reported the synthesis of SWCNTs with a diameter of 1.2 nm by utilizing a carbon–cobalt electrode,^81^ while following a similar procedure, Saito *et al.* and Zhou *et al.* reported the synthesis of SWCNTs using Ni fine particles^84^ and yttrium carbide anode,^85^ respectively. Alternatively, the synthesis of DWCNTs was firstly reported by Hutchison *et al.* using a graphite rod filled with Fe, Co, Ni and S.^86^ However, the quality and purity have become the main issues in the arc discharge method.

Another method for the synthesis of CNTs with high quality and purity is the laser ablation method, which was firstly introduced by Smalley *et al.* in 1995.^87^ In this method, a laser pulse is focused on a graphite–metal composite material as an energy source. Zhang *et al.* reported the synthesis of SWCNTs by applying continuous wave CO_2_ ablation. In this work, they showed that increasing the laser power also enhanced the diameter of the SWCNTs. Bonaccorso *et al.* synthesized MWCNT thin films on alumina substrates by using the pulse laser deposition (PLD) technique with an Nd: YAG laser to introduce the commercially viability of MWCNTs produced thorough the laser ablation method.^88^

In addition to the above-mentioned two routes, CVD is regarded as one of the standard methods for producing ultra-high-quality CNTs. The CVD technique employing by a catalytic system is generally known as catalytic CVD (CCVD).^89^ In addition to CCVD, other routes for the synthesis of CNTs *via* the CVD technique include water-assisted CVD,^90–92^ oxygen assisted CVD,^93^ microwave plasma (MPECVD),^94,95^ radio frequency CVD (RF-CVD)^96^ and hot-filament CVD (HFCVD).^97,98^ Among them, CCVD is regarded as one of the most viable routes for the synthesis of ultra-high-quality CNTs with the option of mass scalability. Fe, Ni, and Co are the most versatile catalysts used in the CCVD technique.^99^ Alternatively, the precursor molecules used in CCVD include hydrocarbons such as methane,^100^ ethane,^101^ ethylene,^102^ acetylene,^103^ xylene,^104,105^ ethanol,^106,107^ isobutane^108^ and their mixtures.^109^ Generally, the substrates used in CCVD are Si, SiO_2_, Ni, Cu, Cu/Ti/Si, stainless steel, and glass, while substrates of tungsten foil and graphite are also employed in some studies.^110,111^ Zhu *et al.* reported the synthesis of uniform crystal-like DWCNTs on a metal catalytic system of Fe and Co on mesoporous silica.^112^ Hiraoka *et al.* reported the CCVD of acetylene over uniformly dispersed metal particles using heat-resistant zeolite-based substrates at a temperature above 900 °C to produce high-quality DWCNTs.^113^ It has been reported that the choice of metal catalyst greatly affects the growth of CNTs.^114^ Additionally, the plasma-enhanced chemical vapour deposition (PECVD) is another method for the production of CNT based hybrid materials for various applications. PECVD is assisted using several different methods such as direct current (DC-PECVD), radiofrequency (RF-PECVD), diffusion (DPECVD) and microwave (MWPECVD). The routes for the synthesis of CNT were widely reviewed by Lim *et al.*, showing the consistency of the PECVD method for the synthesis of CNTs. They reported that the low-temperature synthesis of SWCNTs could be achieved by using the PECVD technique.^115^ Other methods for the synthesis of CNTs include liquid pyrolysis techniques and aerosol pyrolysis process. These are catalytic CVD-based methods, which incorporate the pyrolysis of both liquid catalytic precursors and liquid hydrocarbon. In this regard, Byeon *et al.* reported a new aerosol-assisted chemical vapor deposition (AACVD) process to synthesize high-quality vertically aligned CNTs with an *in situ* configuration of metal nanoparticles in a very short time of 20 min *via* the pyrolysis of ferrocene–ethanol aerosol.^116^

However, although the synthesis of CNTs using these routes shows promise for various applications, low-cost and mass-scale productive techniques still need to be explored. Therefore, to develop cost-effective and scalable methods for the synthesis of CNTs, researchers are searching for low-cost precursor molecules. Fortunately, due to the high carbon content in various carbon-containing waste materials, they show a promising way to produce various types of carbon nanomaterials. In the next section, we show the various routes for the synthesis of carbon nanomaterials using carbon-containing waste materials.

### 3.3 Carbon-containing waste-derived graphene and CNTs

Over the past few decades, solid waste management has become an issue worldwide. With the rapidly increasing population, the solid waste density has risen exponentially in recent years. Especially, developing countries are facing an alarming situation of solid waste, and thereby high-level solutions are necessary to address the problem of solid waste. Solid waste mainly contains two types of waste materials, *i.e.*, bio-degradable waste and non-biodegradable waste. Generally, biodegradable waste materials include waste materials that can deteriorate under environmental conditions, and therefore are less harmful given that most of them originate from natural sources. Agricultural waste and food waste are the two most common examples of bio-degradable waste. In contrast, non-biodegradable waste is often regarded as manmade materials and does not deteriorate under environmental conditions or may take a few hundred years to completely deteriorate. Generally, synthetic polymeric materials such as various types of plastics are categorized as non-biodegradable waste. Currently, the production of polymers and their utilization are increasing rapidly to improve the lifestyle of human beings. However, these polymeric products create several issues in the environment and ecosystem. Although several routes have been identified for the recycling of these polymeric materials, such as the conversion of post-consumer polymers into new types of polymer products through mechanical recycling, conversion of polymeric waste into thermal energy through combustion using the thermal recycling approach and conversion into various types of chemicals and fuels using the chemical recycling approach. However, all these recycling approaches consume more energy and resources than the cost of the recycled product. Therefore, researchers have suggested various other routes to convert trash to treasure. The upcycling of polymeric waste is one of these routes, which can produce value-added products with greater economic benefits. Fortunately, the high carbon content in waste polymeric products opens a new window for the upcycling process to obtain high value-added products with more economic value. The most common polymers such as polyethylene (PE), polypropylene (PP), polystyrene (PS), polyethylene terephthalate (PET) and polyacrylonitrile (PAN) possess about 85.6 wt%, 85.6 wt%, 92.2 wt%, 62.6 wt% and 67.9 wt% of carbon, respectively. The upcycling of these polymeric products includes the production of light hydrocarbons,^117,118^ activated carbon,^119^ carbon fibers,^120,121^ fullerenes,^122,123^ graphene^124^ and carbon nanotubes.^125,126^ Because of the high demand for carbon nanomaterials for various applications in daily life, the upcycling of waste materials to get a bulk amount of carbon nanomaterials can become a suitable choice. Especially, the upcycling of waste plastics into carbon nanomaterials can lead to a dramatic change from the point of ecology and economy. By using waste plastic as a low-cost feedstock material, one-dimensional carbon nanomaterials can be produced in bulk. The first concept of synthesizing CNTs from solid polymeric materials was developed about 24 years ago.^127–130^ Since then, the advancements in the routes for the synthesis of CNTs from waste plastic have shown that there are two possible ways to achieve this, as follows: (1) one-pot synthesis of CNTs from waste plastics and (2) stepwise synthesis of CNTs from waste plastics. The one-pot synthesis of CNTs generally includes the *in situ* formation of CNTs by the developed carbon feedstock of waste plastics, while the stepwise synthesis of CNTs from waste plastic occurs after the development of the carbon feedstock. Typically, the one-pot synthesis of CNTs is initiated with the selection of catalysts or degradation agents, which are properly mixed with the solid waste polymers in a fixed ratio. Subsequently, the catalyst and solid waste polymer mixture is subjected to pyrolysis at a certain temperature, which varies depending on the polymer. Then, the catalytic decomposition of the solid waste polymer is initiated by the pyrolysis process, producing liquid or gaseous phases of lower hydrocarbons, which can serve as carbon sources for the growth of CNTs on the catalysts. Various types of polymers such as PE,^131–134^ PP,^135–139^ PS,^140^ PET,^141^ polyvinyl alcohol (PVA),^142^ polyvinylchloride (PVC)^143^ and phenol-formaldehyde (PF)^144^ have been studied to obtain CNTs, while various types of catalysts have also been investigated such as Ni,^131,145,146,160^ Fe,^140,147,154^ NiO,^148–151^ Ni_2_O_3_ ^159^ ferrocene,^132,152^ ferrous chloride,^153^ and cobalt acetate^133^ and heat is generally supplied by using fixed beds, *i.e.*, electric furnace,^137,151^ autoclave,^109^ combustion of fuels,^136,148,150,155^ and fluidized beds.^135,141^ To evaluate the possible mechanism of the synthesis of CNT *via* one-pot synthesis, Jiang *et al.* proposed a mechanism based on the formation of active intermediates such as carbenium ions during the synthesis of CNTs using PP as the feedstock and an Ni-based catalyst.^155^ This study suggested that the degradation of plastics in the presence of a catalyst occurs *via* the formation of active intermediates, while non-catalytic degradation occurs *via* the formation of free radicals, which play a major role in the formation of CNTs. Further, the synthesis of CNTs is greatly affected by incorporating solid acids such as organically modified montmorillonite (OMMT) or zeolite with a metal catalyst^148,150,152^ and chlorinated compounds such as CuCl and FeCl_3_.^156,158^ The incorporation of solid acids assists the degradation of the molecular chains of the plastics by providing intermediate proton acidic sites, thereby enhancing the rate of the formation of CNTs^155,157^ Additionally, activated carbon together with a metal catalyst enhances the rate of the formation of CNTs *via* three main routes, as follows: (1) it absorbs the radical fragment during plastic degradation, (2) promotes the formation of aromatic compounds, and (3) assists the process of dehydrogenization.^122^

In addition to the different types of polymers and catalysts, physical parameters such as temperature, composition/concentration, reaction time, and inert gas flow also affect the quality and yield of the CNTs. A group of researchers synthesized CNTs from PE employing ferrocene and MAPP as the catalyst at the temperature of 700 °C in an autoclave system,^132^ while Pol *et al.* demonstrated a solvent free route for the synthesis of multi walled carbon nanotubes from LDPE and HDPE by using thermal dissociation approach in presence of chemical catalyst such as cobalt acetate.^133^ Arena *et al.* demonstrated the synthesis of CNTs using a mixture of PP/PE/PET as the carbon precursor, while using alumina particles as a catalyst in a fluidized bed, and heated the system in the temperature range of 450 °C to 850 °C using an electric furnace.^135^ Luo *et al.* synthesized CNTs from PVC using ferrocene as a catalyst for degrading the PVC molecular chains. This group showed the synthesis of CNTs by simultaneously using three reactors. Firstly, they sublimated ferrocene in the first reactor to convert it to gaseous form, and then passed this gas into the second reactor, where the pyrolysis of PVC occurred at a temperature of 800 °C for 12 min. Finally, the carbon feedstock/catalyst mixtures were passed into the third reactor for the complete formation of CNTs, thus resulting in the stepwise synthesis of CNTs.^136^ Similarly, Liu *et al.* reported a two-step process for the synthesis of CNTs using PP as the precursor. Firstly, PP was pyrolyzed in the presence of H-ZSM-5 zeolite in a screw kiln reactor to develop the feedstock of pyrolysis gases, which subsequently decomposed over a nickel-based catalyst in a moving bed reactor system at the optimum temperature of 700 °C.^136^

Although both the one-pot synthesis and multi-step synthesis can be employed for upcycling waste plastic by converting them into CNTs, there are several challenges that must be addressed to obtain a uniform product. One of the major challenges is the supply of waste feedstock, which possesses a lack of consistency in terms of impurities and composition. Given that real-world waste plastic comes in the form of a mixture of polymers, it is a big task to maintain the quality of the final products. Another challenge is the complexity of the processes, which still needs several scientific investigations to make the upcycling process more viable by producing high-quality CNTs.

Besides the synthesis of CNTs, waste polymers have also been explored as precursor molecules for the synthesis of high-quality graphene sheets for various real-field applications. Because of the high carbon content in polymeric waste materials, as discussed earlier, they have been employed for the synthesis of graphene sheets by various researchers. The first approach towards the synthesis of graphene from plastic waste was demonstrated by Tour's group. This group reported the preparation of high-quality and large-area graphene sheets using PMMA, poly(acrylonitrile-*co*-butadiene-*co*-styrene), and polystyrene (PS) on a Cu or Ni substrate at the temperature of 800 °C.^161–163^ Li *et al.* demonstrated the low-temperature CVD synthesis of graphene sheets at the temperature of 400 °C by using PMMA and PS as precursor molecules.^164^ Sharma *et al.* reported the synthesis of single-crystal graphene using an ambient-pressure CVD process, while employing solid waste plastics as the precursor and polycrystalline Cu foil as the substrate.^165^ Similarly, Cui *et al.* reported the synthesis of high-quality graphene sheets on Ni substrates using daily plastic waste.^166^ Wan *et al.* reported the synthesis of high-quality graphene with large area *via* the dehydrogenation of polycyclic aromatic hydrocarbons.^167^ Pandey *et al.* reported the synthesis of the graphene nanosheets from plastic waste by using bentonite nanoclay as the degradation agent and demonstrated its application for dye sensitized solar cells and supercapacitors.^168^ Thus, it can be visualized from the literature review that limited research articles are available on the synthesis of graphene from waste plastics, showed that vast and deep research gap is still present, which must be addressed to use waste plastics as a source for the mass-scale production of cost-effective graphene with greener techniques.

In addition to polymeric waste, paper waste is another type of carbon-containing solid waste that can be efficiently used as a precursor for the synthesis of carbon nanomaterials. The literature survey showed that few articles have been reported on the synthesis of graphene from paper waste. Singu *et al.* reported the synthesis of a graphene-type material using paper waste in 2017. This group reported the synthesis of graphene from waste-paper *via* the combustion process for supercapacitor applications. In this process, the carbonization of the waste-paper was done in the presence of urea at the temperature of 850 °C, and subsequently analyzed by XRD and SEM analysis.^169^ Although, this process resulted in the successful synthesis of graphene from paper waste, major characterization data regarding the quality of graphene were missing in their report. Therefore, it will become a good approach to investigate some other or similar routes for the mass-scale production of high-quality graphene sheets from paper waste. Furthermore, there are several other precursor molecules that are treated as waste, but contain a huge amount of carbon, and therefore may also be treated as a precursor for graphene synthesis. Textile waste is one of these types of waste materials that can be treated as a precursor for the synthesis of graphene sheets or graphene nanofibers. Presently, carbon-containing waste materials are the main type of waste that highly affects the ecology and economy. The literature shows that plastic waste is at the top of carbon-containing waste materials, which can be resolved *via* the upcycling process. Subsequently, the value-added products obtained after the upcycling process can be used for energy harvesting applications.

## 4. Carbon and carbon nanomaterials for PSCs

### 4.1 Carbon-based electrodes for PSCs

Among the various parts of PSCs, the choice of electrode materials has become a serious concern, given that the long-term durability and cost-effectiveness of PSCs also depend on the type of electrodes. Generally, noble metals such as gold- and silver-based electrode materials are used as the back electrode for PSCs. However, the utilization of expensive metals in PSCs hinder their commercialization due to their high cost. Besides, the deposition of these metals requires high energy input through the vacuum evaporation method,^170^ while another drawback associated with the use of these metals is the formation of metal halides, which occurs because of the migration of the halide ions from the active layer to the metal-based electrodes. The formation of these unwanted metal halides degrades the device, and hence the PSCs with these metal electrodes show low stability. Alternatively, carbon-based materials show promising advantages to overcome the problems of metal-based electrode systems. One of the advantages of carbon-based materials is that user-friendly electrode deposition techniques can be employed, where the electrode can be fabricated without the use of heavy instrumentation techniques such as thermal evaporation, sputtering and physical vapour deposition. In the next section, we discuss some of the easy deposition techniques for carbon-based back electrodes.

### 4.2 Deposition techniques for carbon electrodes

The deposition of carbon-based paste as an electrode material over a variety of substrates is mainly done *via* the doctor blade or screen-printing technique. The general configuration for the deposition of suitable electrodes for PSCs requires a transparent conducting substrate, on which a layer of insulator is deposited. Subsequently, the insulator layer is covered with another layer of carbon paste. After the deposition of the carbon layer, a sintering process is generally conducted to create the full electrode system, which is suitable for the infiltration of the perovskite ink. Finally, perovskite ink is drop casted to make full PSCs. Thus, this process for the fabrication of electrodes for PSCs shows that the deposition of the electrode is generally temperature dependent. Thus, the first process in deposition generally requires a high temperature in the range of 400–500 °C, in which a mesoporous layer of carbon paste is deposited on an insulating layer of aluminium trioxide (Al_2_O_3_), zirconium dioxide (ZrO_2_) or titanium dioxide (TiO_2_),^171–175^ where TiO_2_ can be used as a compact layer and mesoporous layer. Generally, the insulator layer is employed to prevent contact between the front and back electrodes, thus avoiding photocurrent leakage. Among the insulators, ZrO_2_ provides larger pores in comparison to Al_2_O_3_ and TiO_2_, and thus facilitates the efficient infiltration of perovskite ink for the proper development of the perovskite phase. Hence, ZrO_2_ is also regarded as the best insulating material for the development of carbon-based electrodes.^173^ The thickness of the insulating layer significantly affects the performance of these electrodes. Liu *et al.* showed that the optimal thickness of ZrO_2_ should be 1 μm,^172^ while Barichello *et al.* demonstrated that it should be 1.8 μm for Al_2_O_3_.^174^ Further, researchers showed that the thickness of the insulating layers should not exceed from the limit of the carrier diffusion length.^176^ Thus, mesoscopic PSCs are generally fabricated using this method. Also, the deposition process requires high temperature for the sintering process, and thus seems to be unsuitable for the deposition of flexible electrodes. Generally, the deposition of flexible substrates requires low temperature, and thus another process for the deposition of carbon-based electrodes at low temperature *via* layer-by-layer deposition has been shown to be promising approach, where carbon-based materials are screen printed or doctor bladed on the top of the HTM or simply perovskite layer. Further, this method also gives flexibility to deposit the carbon layer on another substrate, and then transfer it to a cell. Thus, deposition using this method looks simple, but often suffers from contact problems.^177–179^

These two deposition methods have been extensively explored in recent years. High temperature- and low temperature-processed graphite and carbon black electrodes were investigated and shown to be state-of-the-art for the fabrication of electrodes. In the following sub-sections, we review different types of carbon compositions, temperatures, and fabrication techniques.

### 4.3 High temperature-processed graphite and carbon-black electrodes

**Fig. 3:**
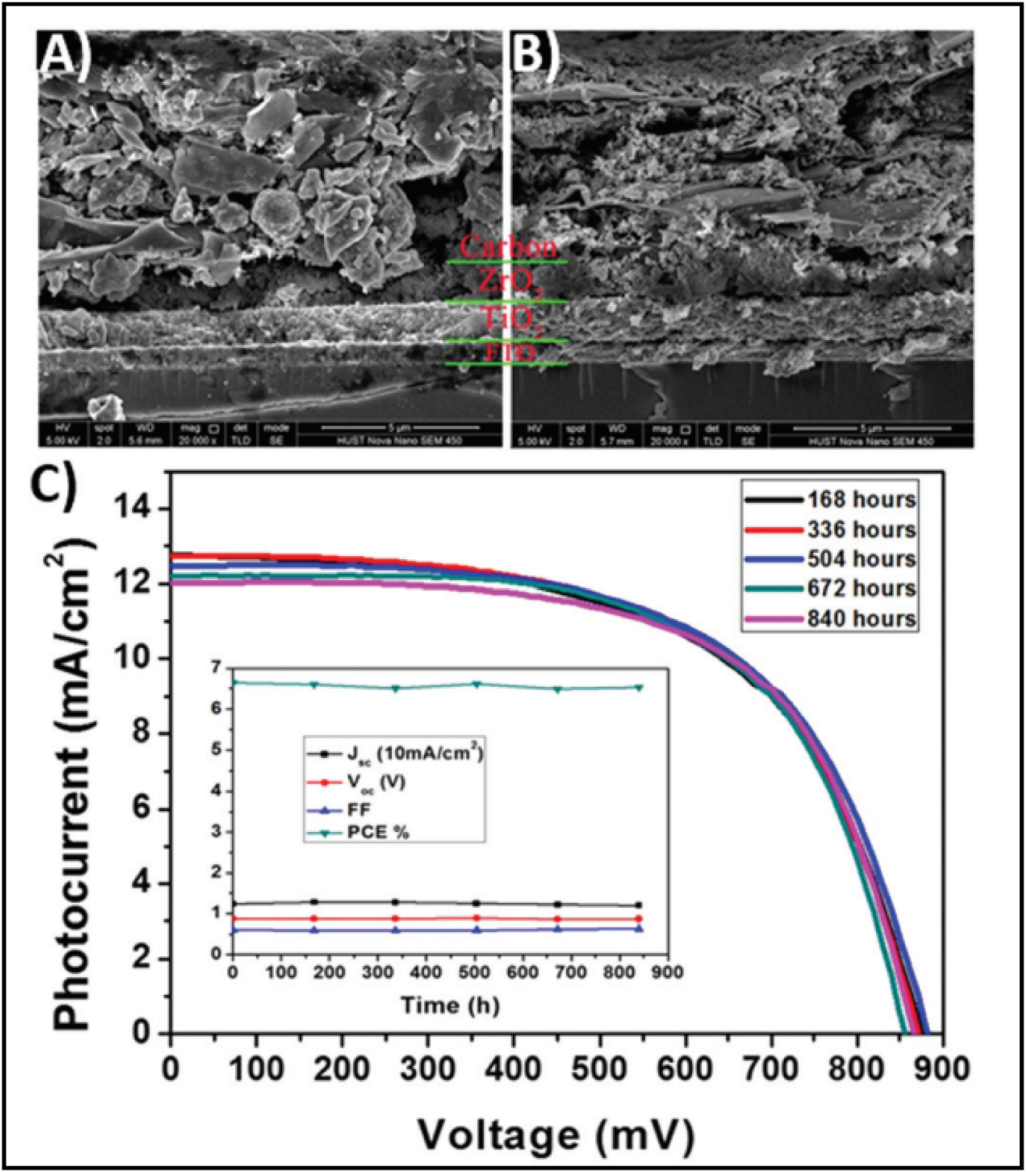
SEM cross sectional images of (A) spheroidal graphite-based device and (B) flake/bulk graphite-based device. (C) Stability analysis graph of the device in the dark at room temperature [reproduced from ref. 180 with permission from Springer Nature, Copyright 2013].

The high conductivity of graphite and porous behaviour of carbon black present a new way for the fabrication of carbon-based electrodes. The first use of graphite and carbon black as back electrodes was demonstrated in 2013 by Ku *et al.*, where they demonstrated a high stability and good PCE with a graphite/carbon black-based composite material for HTM-free PSCs.^180^ Further, to improve the device parameters, spheroidal graphite was used on the top of the carbon composite to enhance its conductivity and surface morphology. The cross-sectional surface electron microscopy (SEM) images showed that the use of the spheroidal graphite improved the surface morphology by filling the surface pores, and thus created a smoother surface (Fig. 3(A) and (B)). Additionally, the introduction of spheroidal graphite improved the PCE from 4.08% to 6.64% and the stability of the device by providing a hydrophobic surface for the prevention of contact with water molecules. Fig. 3(C) shows the stability studies of the as-fabricated carbon black-based monolithic methyl ammonium lead iodide/TiO_2_-based solar cells for long-term monitoring of 840 h stored in dry ambient conditions without encapsulation. Only a slight change was seen in *V*_oc_, *J*_sc_, and FF. Hence, the devices maintained their stability for a longer period and demonstrated nearly 6.5% efficiency even after 840 h. This study clearly reveals that carbon-based PSCs not only provide excellent stability, but also a user-friendly fabrication technique, which can be implemented for the large-scale fabrication of PSCs.

Further, it is worth noting that thickness of the carbon layer also determines the device performance. Zang *et al.* demonstrated that the thickness of the carbon layer may be in the range of 5 μm to 15 μm, but the thickness of 9 μm was found to be the best for optimizing the device parameters. Carbon layer greater than 5 μm exhibited poor conductivity, while that of more than 15 μm thick hindered the penetration of the perovskite precursors, thus resulting in a poor device performance. Further, this group demonstrated that the size of graphite also affects the device parameters. Accordingly, graphite flakes with the size of 8 μm provided bigger pores for the infiltration of the perovskite precursor, and thus helped in the development of a good perovskite phase for efficient PSCs.^181^ This fact was again supported by the study by Raminafshar *et al.*, who demonstrated the effect of the thickness of the carbon layer on the PCEs. This group kept the thickness of TiO_2_ and ZrO_2_ constant, while varying the thickness of the carbon layer to 6.5, 15, 25 and 54 μm. Among them, the carbon film with a thickness of 54 μm showed the poorest PCE of 4.3% due to the hindrance of the infiltration of the perovskite ink, while that with the thickness of 25 μm showed best PCE of 10.7%.^182^

**Fig. 4:**
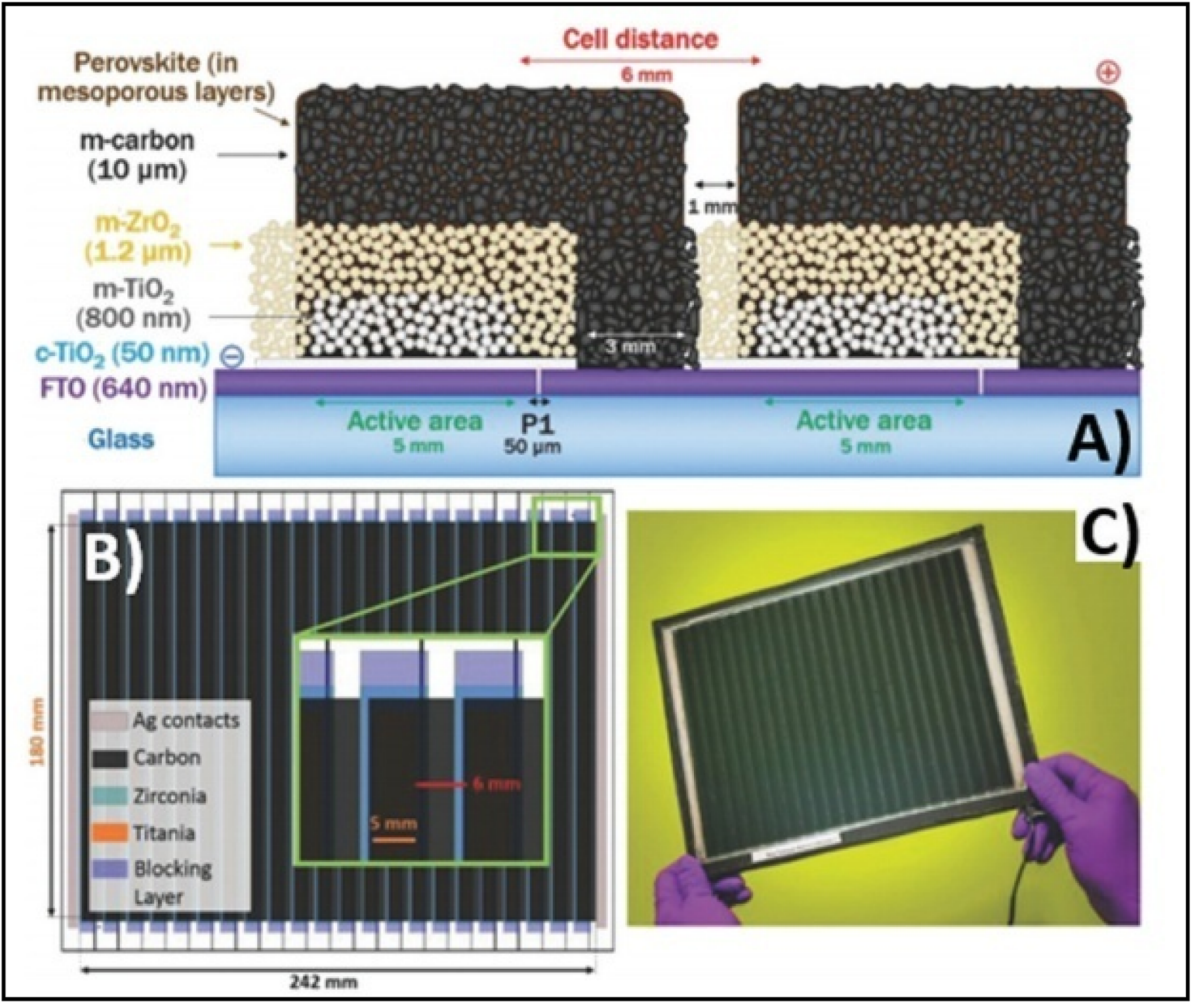
(A) Schematic representation of the module configuration with the dimensions of each component of the module. (B) Schematic of the modules demonstrating the overlapping of the different layers, active area for each cell and module, respectively, and the distance between each cell with a pictorial representation of the actual module. (C) Photograph of the module [reproduced from ref. 188 with permission from the American Chemical Society, Copyright 2015].

Temperature is regarded as another factor that greatly affects the performance of the electrode in PSCs. Annealing carried out at different temperatures resulted in diverse device parameters. Mishra *et al.* demonstrated the effect of the annealing temperature on commercially available carbon-based composite materials for electrode applications. This group deposited carbon paste *via* the screen printing technique and found that high temperature sintering process led to better contact between the carbon particles, and hence allowed excellent passage of current through high conductivity. The electrodes sintered below the temperature of 300 °C showed a poor performance, and hence did not demonstrate good device results due the poor infiltration ability of the perovskite precursors. However, the sample electrodes treated at 350 °C and 400 °C showed the best PCE of 8.4% and 12.4% due to the enhanced uniformity with defect-free morphology of the carbon films, as depicted by the SEM images.^183^ Further, the residual oxygen functionalities also affect the performance of the electrodes in PSCs. Tian *et al.* demonstrated the effect of the oxygen functionalities in carbon-based electrodes.^184^ This group prepared two carbon electrode samples, where one of carbon film possessed a high content of oxygen functionalities with a high surface area of 186.4 m^2^ g^−1^, while the other carbon film possessed a low content of oxygen functionalities with a surface area of 112.5 m^2^ g^−1^. Their studies showed that oxygen-rich carbon (ORC) elevated the work function of the carbon electrode, as well as improved the interface contact between the carbon electrode and perovskite phase. The ORC-based devices also showed a higher PCE of 15.7% that than of the oxygen-deficient carbon (ODC)-based devices, with a PCE of 13.6%. Further, a faster photocurrent response and low hysteresis effect were also observed for the ORC-based devices. Mali *et al.* demonstrated the synthesis of carbon nanoparticles from *Aloe vera* plant at the high temperature of 1000 °C.^185^ The as-derived *Aloe vera* carbon nanoparticles (AV-C) were employed for the fabrication of electrode for PSCs. The AV-C based PSCs exhibited a PCE of 12.58% and showed very high stability. The devices with AV-C retained 85% of their initial efficiency, thus showing the utility of high temperature-processed electrode systems for PSCs. However, the particle size of carbon-based materials also affects the efficiency of the devices. Smooth passage of the perovskite precursors through the channels of the carbon films is necessary for the proper development of the perovskite phase. It has been reported that carbon materials with a larger grain size and specific surface area exhibit excellent properties as a good electrode. Duan *et al.* reported for the first time the use of ultra-thin graphite as a cathode material for PSCs. The ultra-thin graphite was prepared *via* mechanical exfoliation from bulk graphite. This group showed that the ultra-thin graphite possessed better infiltration properties than the bulk graphite. The high surface area of the ultra-thin graphite of 202 m^2^ g^−1^ ensured good contact with the perovskite phase. The recorded device parameters for the ultra-thin graphite devices showed a higher fill factor (FF) of 68% and PCE of 14.07% with *J*_sc_ of 22.89 mA cm^−2^, while the PSC with bulk graphite as the cathode material showed an FF of 62%, *J*_sc_ of 22.89 mA cm^−2^ and PCE of 12.63%, showing that ultra-thin graphite with a large surface area shows good properties as electrodes.^186^ Further, to improve the device stability and PCE of carbon-based PSCs, the infiltration process for the perovskite precursor must be improved. One of the techniques to improve the porosity was demonstrated by Tao *et al.*, where they introduced polystyrene spheres (PSs) in the carbon paste, and subsequently annealed it at high temperature.^187^ The SEM images of the as-doped carbon paste with PSs showed its enhanced porosity, thereby resulting in improved device parameters with *V*_oc_ = 782 mV, *J*_sc_ = 9.32 mA cm^−2^, FF = 56% and PCE of 4.10%. Although high temperature-processed back electrodes in PSCs show an average performance in terms of PCE and stability, but sometimes they exhibit poor performances because of the poor uniformity and compactness of both carbon and TiO_2_. Because of these obstacles, it is often difficult to fabricate large-area PSC modules with high PCE and stability. Rossi *et al.* demonstrated for the first time an example of large-area carbon-based PSCs in an A4 size pattern *via* an optimized printing process, where ZrO_2_ (1.5 mm wide) and TiO_2_ (low temperature processed) were used as an insulator and blocking layer, respectively. The active area of the module was calculated to be about 196 cm^2^, which was composed of 22 units spaced by 6 mm, each having dimensions of 5 × 180 mm^2^ (Fig. 4). Further, the doctor blade coating method was used for the fabrication of a 10 μm thick carbon electrode. A PCE of 3.2% was obtained using this large-area carbon-based PSC.^188^

Carbon-based PSCs also showed utility in high temperature climatic areas, where the temperature exceeds the limit of the phase transition temperature of perovskites.^189^ Baranwal *et al.* demonstrated a carbon-based HTM-free PSC, which maintained stability up to 100 °C.^190^ A three-layer printable device was demonstrated with over-sealed and side-sealed configurations. It was shown that the unsealed and over-sealed devices lost their efficiency by 20% within just 30 h, when kept under the temperature of 100 °C. In contrast, the side-sealed configured device showed excellent stability up to 1500 h at the same temperature. This work suggested that sealing is necessary for the protection of the devices to achieve high thermal stability, while over-sealed devices can cause internal decomposition of the absorber materials, hence leading to rapid device degradation.

### 4.4 Low temperature-processed back electrodes

**Fig. 5:**
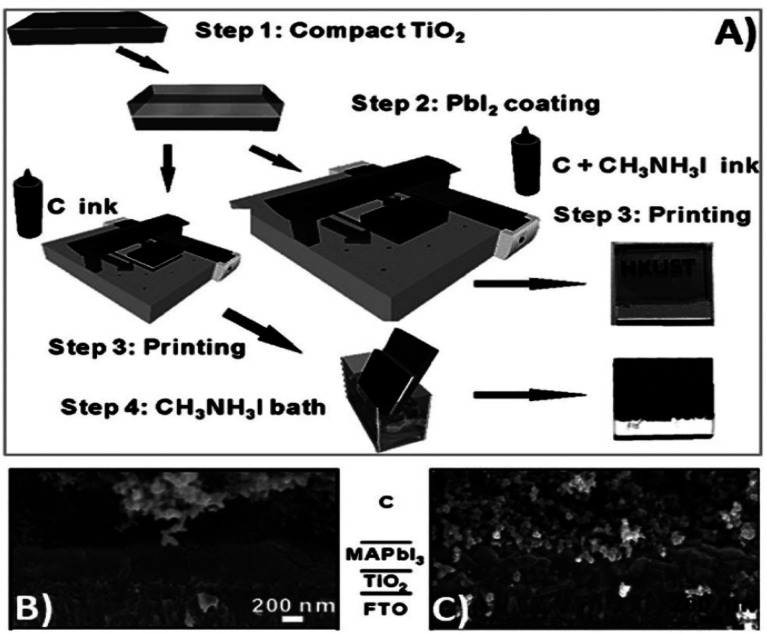
(A) Process for the fabrication of carbon and CH_3_NH_3_I ink-based PSCs by inkjet printing technique, (B) SEM cross sectional image of carbon ink-based TiO_2_/CH_3_NH_3_PbI_3_/carbon solar cells, and (C) SEM cross-sectional image of carbon and CH_3_NH_3_I ink-based TiO_2_/CH_3_NH_3_PbI_3_/carbon solar cells [reproduced from ref. 193 with permission from John Willey & Sons, Copyright 2014].

Generally, high temperature-processed back electrodes require high thermal treatment for their fabrication, involving the use of high energy-consuming equipment. Additionally, the process is time-consuming, and thus requires a long procedure to fabricate this type of electrode system. One of the major problems associated with this electrode system is the non-applicability of its fabrication procedure for flexible substrates, and thus it cannot be employed for the fabrication of large-scale flexible carbon-PSCs.^191^ Hence, low temperature-processed electrode systems have gained special attention because of their cost-effectiveness and time-saving advantages. In this regard, an interesting strategy was demonstrated by Zhou *et al.* in 2004.^192^ This group reported the fabrication of fully solution processable low-cost TiO_2_/CH_3_NH_3_PbI_3_/C heterojunction (HJ) solar cells based on a low temperature-processed carbon electrode. To prepare the carbon electrode, a conductive ink of carbon was firstly prepared by dispersing 5 g of conducting carbon and 4 g of zirconium dioxide pearls in chlorobenzene (15 mL), which was subsequently electro-milled for 2 h. Then, the as-prepared ink was directly coated on the top of CH_3_NH_3_PbI_3_*via* the doctor blade technique, and finally dried at 70 °C for 40 min. Consequently, the complete device showed a PCE of 9.08% and exhibited a very high stability of 2000 h without encapsulation. It was also observed that the charge recombination at the interface of the perovskite phase and carbon must be low. In this regard, Wei *et al.* demonstrated an interesting approach by developing an ink composed of carbon and CH_3_NH_3_I in isopropanol for the fabrication of planer of PSCs.^193^ Subsequently, the developed ink was printed on FTO/TiO_2_/PbI_2_ using an inkjet printer (Fig. 5(A)). This technique not only shortens the time required for the preparation of the perovskite ink, but also deposited the perovskite phase and carbon layer simultaneously with an efficient interfacial contact for smoother charge transportation. To establish the advantages of the present approach, another device was also fabricated, in which a carbon layer was fabricated over FTO/TiO_2_/PbI_2_, which was subsequently soaked in a solution of CH_3_NH_3_I. After the fabrication of both devices, characterization was conducted to analyse the advantages of premixed carbon and CH_3_NH_3_I. The device with the carbon and CH_3_NH_3_I-based ink showed better device parameters than the traditionally fabricated device by developing a better interface between carbon and perovskite, as shown by the SEM images in Fig. 5(B) and (C). The *J*_sc_ improved from 15.00 to 17.20 mA cm^−2^, *V*_oc_ from 0.90 to 0.95 V, FF from 63% to 71%, and finally the PCE from 8.51% to 11.60%. Further, the recombination resistance (*R*_rec_) studies showed that the charge recombination decreased several times for the carbon and CH_3_NH_3_I ink-based PSCs. Also, it was shown that the unsealed champion devices retained almost 90% of their PCE when stored in the dark even 12 days after their fabrication.

**Fig. 6:**
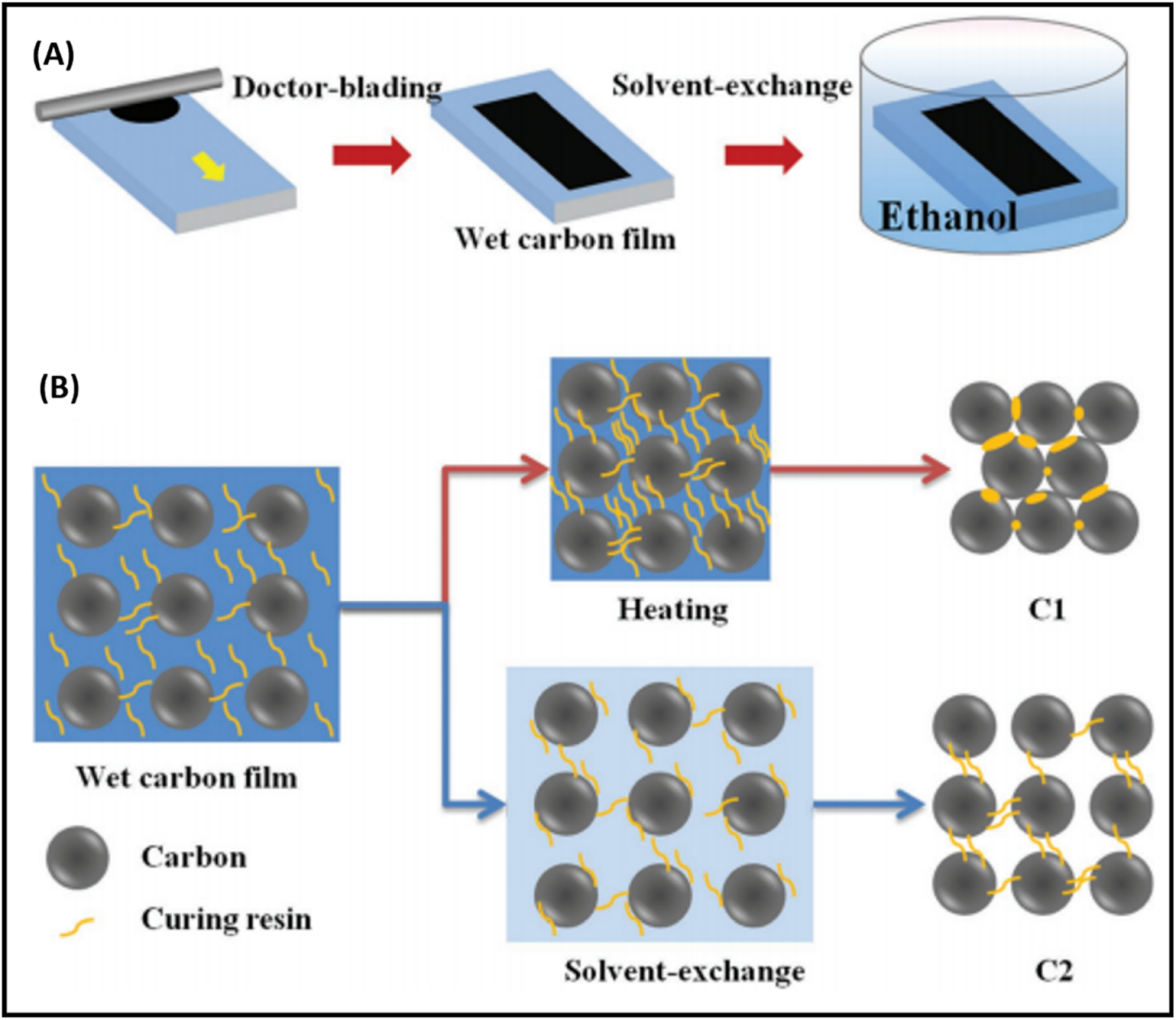
(A) Solvent-exchange method for the preparation of C2 films. (B) Mechanism of microscopic curing for the formation of C1 and C2 films [reproduced from ref. 194 with permission from John Willey & Sons, Copyright 2018].

It has been noted that the interface contact between the carbon and perovskite phase must be strong to achieve remarkable PCEs, especially in the case of low temperature-processed carbon-based PSCs, where the carbon and perovskite phases often suffer from poor interface contacts. Generally, the carbon paste used for the fabrication of electrode system consists of a variety of solvent systems, which, on evaporation, leave pinholes or sometimes swell rapidly. The swollen layer of carbon together with pinholes leads to poor device performances. Thus, to overcome this problem, a possible solution was demonstrated by Zhang *et al.*, where they reported a facile technique for the preparation of a self-adhesive carbon film *via* the solvent exchange method at room temperature.^194^ This group fabricated a carbon electrode, named C2, by applying carbon paste on a glass substrate and soaking it in ethyl alcohol at room temperature (Fig. 6(A)). Because of the solvent exchange process, the carbon film became self-adhesive in nature, and hence easily peeled off from the glass substrate to form a self-standing carbon film with a thickness of 60 μm. Fig. 6(B) shows the microscopic curing mechanism, according to which during the solvent exchange process, the intimate contact of ethanol with the carbon framework suppressed the curing and densification process in the carbon framework. Subsequently, this C2 carbon film as an electrode system was coated over the perovskite layer and compressed to create a good adhesion interface between C2 and the perovskite layer. For comparison, another carbon electrode, named C1, was also fabricated *via* the traditional method, in which carbon paste was deposited on a glass substrate and subsequently heated at 100 °C. It was found that the C2 carbon film exhibited a better interface, contact in comparison to C1, and hence resulted in a better PCE. The PCE for the C2-based PSCs was found to be 19.2%, while it was 15.2% for the C1-based PSC.

In the field of both high temperature- and low temperature-processed carbon electrodes has shown tremendous progress in recent years. Regarding the progress of the fabrication methods for the development of low and high temperature-processed carbon electrodes for PSCs, it can be concluded that strong interfacial contact between the carbon layer and perovskite phase must be established to achieve an efficient device. Further, the sheet resistance, porosity and conductivity of the carbon layer must be maintained during the fabrication of the carbon electrode. Additionally, the size of carbon particles also leads to a significant improvement in the device parameters, and several strategies have been reported, which showed that carbon nanoparticles with an appropriate size may improve the device parameters. Table 2 presents an overview of the different types of fabrication methods for carbon-based electrodes together with the deposition technique, temperature assessment, sheet resistance, thickness, and the obtained PCE by implementing these electrodes.

**Table 2:** Different techniques for the fabrication of carbon back electrodes together with a comparative study of the sheet resistance and thickness of the carbon layer

Composition of paste for electrode fabrication	Technique employed for deposition	Thermal treatment (°C)	Sheet resistance (Ω cm^−2^)	Thickness (μm)	PCE (%)	Ref.
ZrO_2_ (1 g, particle size 30 nm), graphite (6.5 g), carbon black (2 g, particle size 30 nm), hydroxypropyl cellulose (1 g), and terpineol (30 mL)	Screen printing	400 °C, 30 min	8.98	10	14.15	195
Graphite (10 wt%), CB (5 wt%), ethyl cellulose (20 wt%), terpineol (60 wt%)	Screen-printing	125 °C, 10 min; 325 °C, 10 min; 375 °C, 10 min; 400 °C, 30 min	Not reported	4–5	12.12	196
Bulk density graphite (6.5 g), carbon black (2 g), ZrO_2_ (1 g), hydroxypropyl cellulose (1 g), terpineol (30 mL)	Screen-printing	400 °C, 30 min	16	9.6	13.6	197
Graphite (6.5 g), carbon black (2 g), ZrO_2_ (1 g), hydroxypropyl cellulose (1 g), terpineol (30 mL)	Screen-printing	400 °C, 30 min	35	11	12.4	197
Graphite (2.8 g), carbon black (0.7 g), ZrO_2_ (0.42 g), polystyrene spheres (1.68 g) in ethanol	Screen-printing	400 °C, 60 min	180.7	Not reported	3.13	198
Graphite (3.6 g), carbon black (0.9 g), ZrO_2_ (0.54 g), polystyrene spheres (0.56 g) in ethanol	Screen-printing	400 °C, 60 min	68.3	Not reported	4.10	198
Graphite (3.8 g), carbon black (0.95 g), ZrO_2_ (0.57 g), polystyrene spheres (0.28 g) in ethanol	Screen-printing	400 °C, 60 min	56.7	Not reported	3.87	198
Graphite (4 g), carbon black (1 g), ZrO_2_ (0.6 g) in ethanol	Screen-printing	400 °C, 60 min	30.5	Not reported	3.36	198
Graphite : carbon black = 3 : 7, hydroxypropyl cellulose, terpineol	Screen-printing	400 °C, 30 min	23	11	15.70	199
Ultra-thin graphite, carbon black, hydroxypropyl cellulose, terpineol	Printing	400 °C, 30 min	5–25	5–16	14.01	199
Bulk graphite, carbon black, hydroxypropyl cellulose, terpineol	Printing	400 °C, 30 min	5–20	5–16	12.63	199
Graphite (5 g), carbon black (1 g), ZrO_2_ (1 g), terpineol (30 mL)	Blade coating	400 °C, 30 min	Not reported	Not reported	13.7	200
Graphite (9 g), carbon black (3 g), ZrO_2_ (1 g), hydroxypropyl cellulose (15 g), terpineol (18 g)	Screen-printing	400 °C, 30 min	56	25	10.7	201
Carbon black (15 mg mL^−1^ in iso-propanol)	Screen-printing	100 °C, 60 min	Not reported	Not reported	7.55	202
Carbon black (15 mg mL^−1^ in iso-propanol), CH_3_NH_3_I (10 mg mL^−1^) in 2-isopropanol	Screen-printing	100 °C, 60 min	Not reported	Not reported	10.03	202
Polyvinyl acetate (20 wt%), carbon material (80 wt% of graphite : CB = 1 : 0)	Doctor blade	85 °C, 15 s	1.11	40	10.27	203
Polyvinyl acetate (20 wt%), carbon material (80 wt% of graphite : CB = 5 : 1)	Doctor blade	85 °C, 15 s	0.75	40	11.43	203
Polyvinyl acetate (20 wt%), carbon material (80 wt% of graphite : CB = 3 : 1)	Doctor blade	85 °C, 15 s	0.69	40	13.53	203
Polyvinyl acetate (20 wt%), carbon material (80 wt% of graphite : CB = 2 : 1)	Doctor blade	85 °C, 15 s	0.61	40	12.47	203
Commercially available carbon ink	Blade coating	100 °C, 15 min	Not reported	11.2	11.92	204

### 4.5 Carbon-based transparent conducting electrodes

Carbon-based nanomaterials (CNMs) have gained significant attention in recent years by researchers due to their excellent electrical and optical properties, and consequently identified as alternative candidates to ITO for the preparation of transparent conducting electrodes (TCEs) in solar cell applications. Presently, a wide variety of CNMs has been identified as candidates for TCEs.^205^ CNTs and graphene have both been widely explored in recent years to fabricate highly conducting TCEs. However, due to the tube–tube junction resistance of CNTs, graphene-based TCEs are more favourable for the development of TCEs. ITO-based TCEs are the most used electrodes for solar cell applications due to their low sheet resistance (*R*_sheet_) and high optical transparency. However, the limited availability of indium and high fabrication cost make it an expensive electrode system for solar cells. Alternatively, fluorine-doped tin oxide (FTO)-based electrodes are another class of TCEs widely used in solar cells. However, due to the temperature dependency performance of FTO-based TCEs, their versatile properties cannot be exploited in solar cells. It has been reported that at a higher temperature, FTO-based TCEs show a high sheet resistance, thereby causing current leakage due to the defects present in the surface of FTO. To date, numerous ITO- and FTO-based TCE candidates have been identified such as transparent conducting oxides (TCO),^206^ metal nanowires,^207^ conducting polymers^208^ and carbon nanotubes (CNTs).^209,266^ To evaluate the suitability of these materials, their figure of merit (FOM) is generally evaluated, which gives information about their effectiveness as TCEs. The FOM for TCEs generally depends on both the electrical conductivity and optical transparency of the coated material. Both the electrical conductivity and optical transparency should be balanced in TCEs. Especially, the photovoltaic performance of the devices also depends on the nature of the TCEs. Alternatively, the thickness of the coated material can also affect the FOM value of TCEs. Several formulations have been reported for the calculation of FOM by various researchers.

The first evaluation of the FOM was introduced by Fraser and Cook^210^ in 1972. According to them, the FOM value of TCEs can be calculated using the following expression:

1 FOM = *T*/*R*_s_

where *T* is the transmittance at 500 nm and *R*_s_ is the sheet resistance. A few years later, Haacke^211^ proposed a thickness-dependent formula for the calculation of FOM. According to the Haccke, the FOM value of TCEs can be evaluated using the following expression:

2 FOM = *T*^10^*R*_s_^−1^

where *T* is the transmittance and *R*_s_ is the sheet resistance. Further, another expression for the evaluation of the FOM (thickness independent) was given by Jain and Kulshreshtha,^212^ where according to them, the evaluation of FOM can be done using the following expression:

3 FOM = −*R*_s_ ln *T*

where *T* is transmittance at 500 nm and *R*_s_ is the sheet resistance. Although several other expressions have been derived by various researchers for the evaluation of the FOM, the above-mentioned three expressions seem to be sufficient to calculate the FOM value of TCEs. The industrial demand of any TCE requires an average *R*_sheet_ of 100 ohm per sq. and optical transmittance of >90%, while for solar applications, the *R*_sheet_ should be in the range of 10–20 ohm per sq. and transmittance of >90%. Therefore, efficient materials are required to fulfil these requirements. Recently, a 2D analogue of carbon nanomaterials, *i.e.*, graphene, has shown tremendous interest as a candidate for TCEs. Because of its high electrical and optical properties, graphene is regarded as an alternative candidate for TCEs. However, the quality of graphene sheets depends on their synthesis routes (as discussed in previous sections), which may alter the sheet resistance and optical transmittance of the thin film of graphene sheets. In this regard, Eda *et al.* showed the fabrication of solution-processed thin films of reduced graphene oxide (rGO), depicting *R*_sheet_ of 70k ohm per sq. and transmittance (*T*) of 70% with FOM value of 0.011.^213^ Similarly, Wu *et al.* reported the preparation of solution-processable thin films of graphene oxide, which were subsequently reduced in hydrazine and exhibited an *R*_sheet_ of 100k ohm per sq. and *T* = 95% with FOM value of 0.073.^214^ It has been observed that solution-processable thin films of graphene show a high *R*_sheet_ value in comparison to CVD-based thin films of graphene. Kalita *et al.* reported the preparation of CVD-based graphene thin films with *R*_sheet_ of 1.645 K ohm per sq. and *T* = 81% with an FOM value of 1.031.^215^ The same group also reported the preparation of CVD-based thin films of graphene with an *R*_sheet_ of 860 ohm per sq. and *T* = 81% with an FOM value of 1.031.^216^ Cha *et al.* reported the fabrication of monolayer graphene films synthesized *via* the CVD method with a low *R*_sheet_ of 650 ohm per sq. and high *T* = 97% with an FOM value of 18.897.^217^ Gunes *et al.* reported the CVD synthesis and thin film fabrication of 4-layer graphene with an *R*_sheet_ of 725 ohm per sq. and *T* = 97.6% with a high FOM value of 21.276.^218^ All these methods for the fabrication of graphene-based TCEs showed an optimum range of sheet resistance and optical transmittance; however, the industrial application of these TCEs still cannot meet the required range of *R*_sheet_ and *T*%. These problems can be overcome by the doping effect, which significantly improves the *R*_sheet_ without sacrificing the transmittance value. Kim *et al.* reported the effect of AuCl_3_ doping in graphene thin films. The doping effect of AuCl_3_ significantly reduced the *R*_sheet_ = 150 ohm per sq. and *T* = 87% with an FOM value of 17.43,^219^ while Gunes *et al.* reported the doping of AuCl_3_ on 4-layer graphene sheets and achieved a significant *R*_sheet_ of 54 ohm per sq. and optical transmittance of about 85% with FOM value of 41.24.^218^ Further, Kwon *et al.* reported the doping of Au(OH)_3_, Au_2_S, and AuBr_3_ on few-layer graphene sheets and showed the *R*_sheet_ of 820 ohm per sq., 600 ohm per sq. and 530 ohm per sq., with the improved the optical transparencies of 93%, 86% and 95%, respectively. The graphene sheets doped with AuBr_3_ showed the best FOM = 13.69 in comparison to Au(OH)_3_ and Au_2_S having FOM values of 6.221 and 12.03, respectively.^220^ Jang *et al.* reported the doping of HNO_3_ over multilayer graphene and showed the improved *R*_sheet_ of 500 ohm per sq. with high optical transparency of 96%, while the evaluated FOM was found to be 18.28.^221^ In addition to doping in graphene sheets, several researchers also reported the preparation of graphene hybrid films with improved FOM values. In this regard, Yun *et al.* reported the preparation of hybrid films of GO and silver nanowires (AgNWs) and reported a high FOM value of 60.16,^222^ while Lee *et al.* depicted the preparation of hybrid films of graphene and AgNWs, which demonstrated a high FOM value of 181.8.^223^ Significant work on hybrid film technology was demonstrated by Deng *et al.*, where they reported a high FOM value of 206.9 for graphene and AgNW-based hybrid films.^224^ The best FOM values were observed in the case of the graphene hybrid films consisting of metal grid networks. A breakthrough was achieved by the graphene and Cu grid-based hybrid films, where a high FOM value of 348 was demonstrated by Ho *et al.*^225^ The highest value of FOM = 9998 was depicted by graphene and Ag grid-based network, as reported by Gao *et al.*^226^ Other hybrid films of graphene and CNTs were also reported by several researcher groups. One group reported the preparation of hybrid films of rGO and MWCNT and showed a low value of FOM = 0.033,^227^ while the hybrid films of graphene and CNTs showed a high value of FOM = 12.30.^228^

**Fig. 7:**
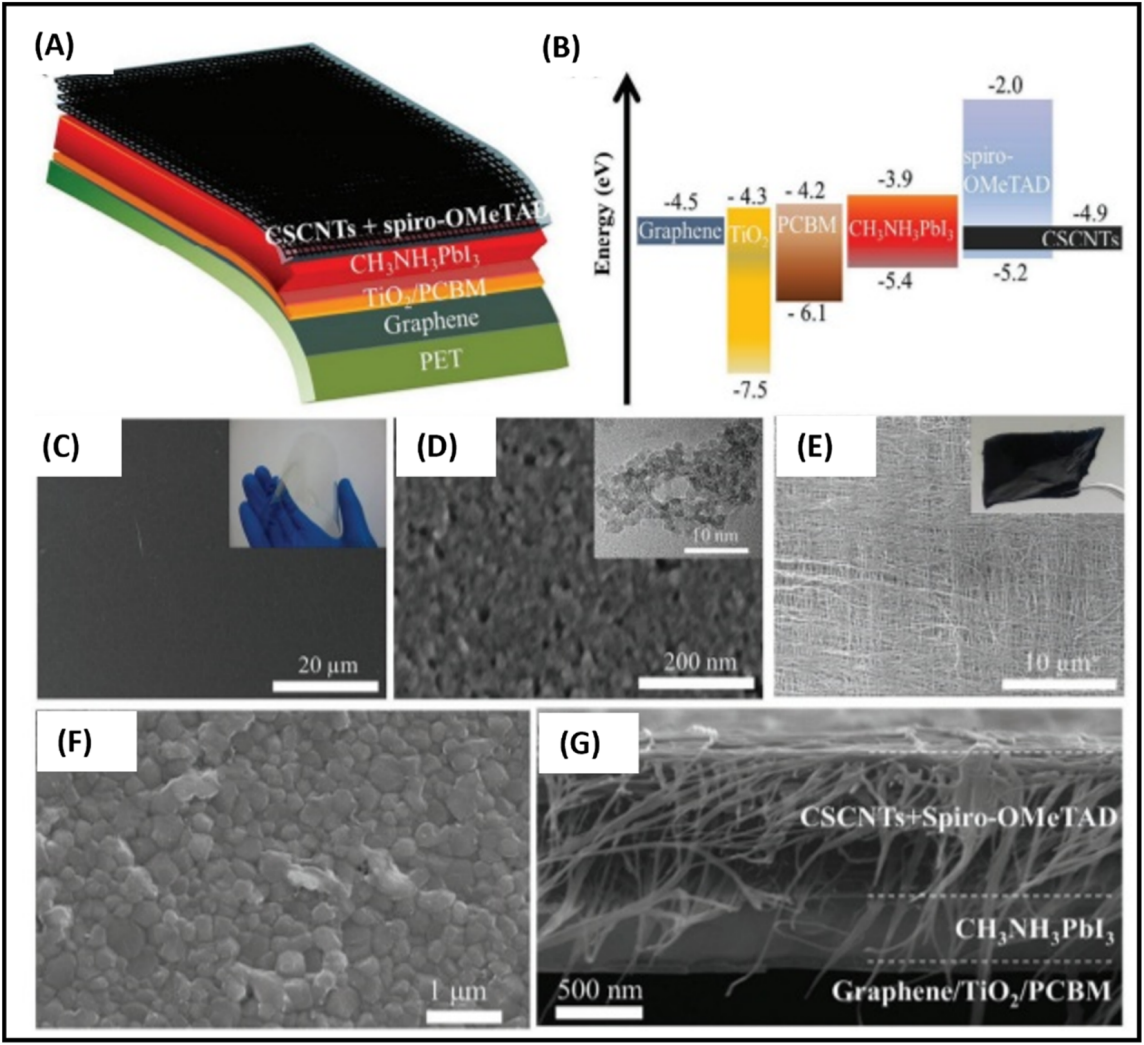
(A) Schematic architecture of the all-carbon-electrode-based PSCs in flexible PET substrate. (B) Energy level diagram of the different layers of the device. (C) SEM image of graphene/PET substrate (inset shows a photograph of PET/graphene TCE). (D) SEM image of TiO_2_ (inset shows TEM image of TiO_2_), (E) SEM image of cross-stacking CNTs (inset shows a photograph of fabricated cross-stacking CNT back electrode), and (F) SEM image of perovskite film, (G) SEM cross-sectional image of all-carbon-electrode-based flexible PSCs [reproduced from ref. 233 with permission from the American Chemical Society, Copyright 2018].

The first use of CVD-processed graphene-based TCEs in inverted PSCs was shown by You *et al.* in 2015.^229^ The transparent layer of graphene (after peeling off from graphene/poly(methyl methacrylate) (PMMA)/poly(dimethylsiloxane) (PDMS)) was deposited over a layer of perovskite/HTM layer. To enhance the hole extraction ability of the graphene layer, poly(3,4-ethylenedioxythiophene)–poly(styrenesulfonate) (PEDOT:PSS) was used as a modifier. Further, the addition of d-sorbitol to PEDOT:PSS enhanced the contact efficiency of the graphene layer with an HTM, which can be seen by the difference in PCE. The PCE significantly improved from 4.13% to 12.37% when the illumination was done from the FTO side. The same results were found when the illumination was done from the graphene side. This work depicted that the PCE was greatly affected by the number of graphene layers in graphene-based TCEs. A PCE of 12.37% was found for bilayer graphene, while a single-layer graphene-based TCE depicted a PCE of 9.18%. However, an enhancement in the number of layers of graphene resulted in a slight reduction in the PCE. The device with three-layer graphene depicted a PCE of 11.45%, while that with four-layer graphene depicted a PCE of 11.27%. Accordingly, the transmittance of the electrode was greatly affected with an increase in the number of graphene layers, which resulted in a poor device performance. Further, the contact between the HTM and back electrode must be strong to realize a better device performance. The hydrophobic nature of graphene causes it to show low affinity for PEDOT:PSS, hence hindering the smooth contact of PEDOT:PSS with its surface, which causes a reduction in the device performance. One of the solutions to this problem was demonstrated by Sung *et al.*, who demonstrated the use of a graphene/MoO_3_-based transparent cathode for PSCs.^230^ They showed that the addition of MoO_3_ to graphene not only reduced the hydrophobic nature of graphene, but it also ameliorated its work function from 4.23 to 4.71 eV. The champion device with the graphene/MoO_3_-based transparent cathode showed a *V*_oc_ of 1.03 V, *J*_sc_ of 21.9 mA cm^−2^, FF of 72% and high PCE of 16.1%, which showed the efficient ability of the graphene-based TCE for PSCs. Further, Yoon *et al.* demonstrated the efficient doping of MoO_3_ in graphene for the preparation of flexible TCEs.^231^ They used polyethylene naphthalate (PEN) as a plastic substrate to prepare a flexible TCE system of graphene and MoO_3_. The champion devices with graphene/MoO_3_ as the back TCEs exhibited a *V*_oc_ of 0.99 V, *J*_sc_ of 21.0 mA cm^−2^, FF of 72% and PCE of 15.0%. Thus, these works showed that the addition of MoO_3_ enhanced the ohmic contact of the graphene surface with the HTM, and thereby the PSCs showed improved parameters. Further, the high conductivity of graphene-based materials is highly desirable to achieve better ohmic contact. In this regard, Zhu *et al.* demonstrated a facile way to enhance the conductivity of graphene by doping nitrogen atoms in the surface of graphene. The nitrogen-doped graphene (NGF) was used as a cathode material for efficient charge transportation. The enhancement in the PCE from 8.98% for the pristine to 10.32% for the NGF-based devices showed that the lone pair of electrons of the nitrogen atoms significantly enhanced the charge transportation ability of graphene.^232^ Lou *et al.* demonstrated a unique approach for the fabrication of carbon nanomaterial-based PSCs.^233^ This group selected graphene as the anode material because of its elevated transparency, while CNTs were used as the back electrode material due to their high electrical conductivity, as shown in Fig. 7(A). Further, this group demonstrated that graphene and CNTs are both suitable candidate electrode materials, as concluded from the energy level diagram (Fig. 7(B)). Due to the high transparency and flexibility of graphene, a well-flexible TCE on PET substrate was demonstrated with a sheet resistance of 290 ± 17 Ω sq.^−1^ and high transparency of 87.3%. The SEM and TEM analysis of TiO_2_ demonstrated that the anatase phase of TiO_2_ possessed a crystal size of approximately 5 nm. The studies done by this group showed that the alignment of the CNTs was parallel in the same plane, while perpendicular in the neighboring plane (Fig. 7(C)–(E)). Further, it was demonstrated that a tetragonal phase of CH_3_NH_3_PbI_3_ existed on the PET/graphene surface with a crystal size of approximately 500 nm. Finally, the overall SEM cross-sectional images of the all-carbon-electrode-based device depicted the respective thickness of each layer in the flexible PSC (Fig. 7(F) and (G)). The as-fabricated device showed a *J*_sc_ of 20.25 mA cm^−2^, *V*_oc_ of 0.89 V, FF of 65% and good PCE of 11.9%.

Further, due the suitable work functions of CNTs, Jeon *et al.* employed them as the front and back electrodes in PSCs, while PEDOT:PSS and [6,6]-phenyl-C_61_-butyric acid methyl ester (PC_61_BM) were used as the HTM and ETM, respectively.^234^ This group demonstrated the ability of CNTs to work as good electrode materials for both rigid and flexible electrodes. A PCE of 7.1% and 7.3% were obtained for the flexible and rigid substrates, respectively. Thus, this work showed that CNTs can also be used a material for the preparation of TCEs. Li *et al.* demonstrated the use of CNTs as back electrode materials in PSCs and depicted a PCE of 6.87% for the CNT-based PSCs.^235^ This work showed that a strong and good connection between the perovskite phase and CNT film can be realized by the addition of toluene, which induces van der Waals forces between the perovskite and CNTs. The effect of toluene was further shown by PL quenching, which depicted the strong adhesion between the CNT and perovskite film interface. Research showed that MWCNTs can also be used for the enhancement of the device parameters.^236^ The stacking properties of MWCNTs provide a platform to develop a uniform and well-connected network to form homogeneous films, which can be easily attached to the perovskite layer. Zheng *et al.* reported the use of boron-doped MWCNTs (B-MWCNTs) as the back electrode material in PSCs.^237^ This group prepared B-MWCNTs *via* the reaction of boric acid with MWCNTs, which were later thermally treated to obtain B-MWCNTs. Because of the improved charged extraction capability and improved band alignment, the B-MWCNT-based PSC showed improved device parameters with a PCE of 14.60%, while the MWCNT-based PSC depicted an average PCE of 10.70%. This work suggests that the replacement of a few carbon atoms with boron atoms without changing the actual graphitic skeleton successfully enhances the hole extraction ability and charge transportation ability by improving the work function and conductivity of B-MWCNTs. Further, to enhance the hole extraction ability of CNTs, various types of the organic and inorganic HTMs have been investigated.^215,238–240^ Aitola *et al.* demonstrated the use of CNTs as the back electrode in PSCs with the use of spiro-OMeTAD as the HTM, achieving a PCE of 14.3%, while the PSCs with gold as the back electrode depicted a PCE of 18.4%, which is found to be higher than that of the CNT-based PSC.^239^ However, the CNT-based PSCs depicted higher stability in comparison with the gold-based PSCs. The CNT-based device exhibited a low loss percentage of 0.04% in 8 h, while the gold-based device showed a drastic loss of 20% during the same period. This work showed that the strong hydrophobic nature of CNTs endows the PSCs with excellent stability, which was not found in the gold-based PSCs. Liu *et al.* demonstrated the use of nickel oxide (NiO) as the HTM in CNT-based PSCs.^241^ Because of the tuned energy level of NiO and CNTs, the NiO/CNT composites exhibited a similar energy level with that of the perovskite materials, and hence provided an excellent path for efficient hole extraction. Further, it was also depicted that addition of NiO significantly reduced the thickness of the electrode without sacrificing its conductivity. The champion device with a fully printed CNT electrode exhibited a PCE of 12.7%, which was found to be much higher than that of the graphite-based device of 6.2%. Additionally, it has been reported that the incorporation of CNTs, rGO, and other carbon nanoparticles in perovskite ink can improve the crystallization process, and therefore provide a larger perovskite grain size and decrease the grain boundaries to improve the transportation of holes and electrons. The reported PCEs obtained for these materials were 19.5%, 18.8% and 18.3%, respectively.^242–244^ Thus, it can be concluded that carbon-based nanomaterials show excellent properties as electrode materials for a variety of solar cell applications. Especially for PSCs, carbon-based materials show unique properties to improve the various device parameters. These materials can not only be used as TCE materials, but also protect the device from moisture, which significantly enhances the stability of the PSCs. In the next section, we discuss the stability status of carbon-based PSCs.

## 5. Hole transport materials for carbon-PSCs

Although carbon-based PSCs are capable of functioning in the absence of a hole transport material (HTM), previously reported work depicted that use of an HTM can improve the hole extraction ability in carbon-based PSCs. Spiro-OMeTAD is the most commonly used HTM in gold-based PSCs; however, its needs to be doped with lithium bis(trifluoromethanesulfonyl)imide (LiTFSI) to further enhance its conductivity. Furthermore, the high cost of spiro-OMeTAD and the hygroscopic nature of LiTFSI hinder the proper use of this HTM in PSCs. Hence, several other types of HTMs have been investigated in recent years, including inorganic, organic and polymeric HTMs.^245–251^ Nickle oxide (NiO), copper sulphide (CuS) and copper thiocynide (CuSCN) are the most used inorganic p-type HTMs, which were recently used in PSCs.^249–251^ The addition of CuS as an HTM in PSCs was demonstrated for the first time in 2018.^252^ Continuing this work, Hu *et al.* reported CuS as an HTM material in carbon-based PSCs, where the low temperature precipitation method was employed for the preparation of undersized CuS particles.^249^ Subsequently, these small CuS particles were mixed with carbon paste in a ratio of 0.5–2 wt% and used to fabricate a back electrode with a thickness of 10 μm. The *J*–*V* characteristics showed that partial doping of CuS enhanced the PCE by 21%, which can be seen from the enhancement in the PCE value from 8.41% to 9.32% for 0.5%-doped CuS, while it was 10.22% for 1%-doped CuS.

Lv *et al.* and Mashhoun *et al.* reported the use of CuSCN as an efficient p-type HTM for carbon-based PSCs.^245,250^ It was found that the incorporation of CuSCN demonstrated an easy solution processable fabrication technique along with the ability of band alignment properties with absorber materials. The group of Lv *et al.* spin-coated a 300 nm-thick CuSCN layer with perovskite layer and found that CuSCN established better contact between the perovskite phase and carbon back electrode. Photoluminescence (PL) studies showed an enhanced emission quenching effect for the perovskite phase, which again confirmed the effective contact and ameliorated band alignment with the perovskite film. The *J*–*V* characteristics of the CuSCN HTM-based device showed that the device parameters significantly improved. The *V*_oc_ was enhanced from 0.72 to 0.78 V, *J*_sc_ from 17.32 to 19.58 mA cm^−2^, FF from 53% to 59%, and ultimately the PCE increased from 6.61% to 9.01%. For the same HTM, the group of Mashhoun *et al.* reported a PCE of 8.59%, which showed that CuSCN can be used as a cost-effective solution-processable HTM for PSCs. However, the device stability studies showed that these devices still need modifications to achieve a balance between PCE and stability. In the last few years, NiO has been identified as one of the widely used inorganic HTM in PSCs. The electron-blocking ability and smooth hole conduction properties of NiO make it suitable as an HTM. Further, it has been reported that NiO reduces the process of recombination, and thereby enhances both the *J*_sc_ and *V*_oc_. The use of NiO as an HTM in low temperature-processed carbon-based PSCs was depicted for the first time in 2017 by Peiris *et al.*^251^ A modified one-step fabrication process was demonstrated in a nitrogen atmosphere with the architecture of FTO/TiO_2_/ZrO_2_/NiO/perovskite/carbon, where the carbon film was deposited at low temperature *via* the doctor blade technique on top of NiO. It was reported that the addition of NiO enhanced the crystallization of perovskite, and hence effectively assisted the process of charge extraction and transfer. Further, the recombination resistance was found to be stronger when NiO was used as the HTM in the devices, which confirmed the suppression of charge recombination. It was found that the PCE significantly improved from 5.96% to 10.35%, which showed a drastic change in the PCE by using NiO as the HTM. Alternatively, low-cost conducting organic polymers such as poly(3-hexylthiophene) (P3HT) also possess the capability of better hole conduction in carbon-based PSCs. Mashhoun *et al.* demonstrated the utility of P3HT as an HTM in carbon-based PSCs by using different types of solvent systems.^245^ This group demonstrated that the choice of the solvent system for P3HT also affects the device parameters. Three solvent systems were selected to dissolve P3HT, namely, chlorobenzene, xylene, and toluene. It was found that P3HT showed the optimum behavior with toluene, while chlorobenzene and xylene showed a lower performance for dissolving P3HT, and hence the devices with these solvents showed a poor performance. A PCE of 1.27% and 3.45% was obtained for the chlorobenzene- and xylene-based devices, respectively, while the toluene-based devices showed a high PCE of 5.04%. Additionally, this group also depicted that a good contact between P3HT and carbon can again improve the device performance. This was done by incorporating TaWO_*x*_ nanoparticles (NPs) between P3HT and carbon. It was found that the PCE of the device drastically increased from 5.04% to 11.58%, while the *V*_oc_ of the device was also enhanced from 0.764 V to 1.012 V. Thus, this research showed that good contact is necessary to achieve the optimum result by using P3HT as the HTM. Good contact must be established between P3HT and carbon, which can be done *via* interfacial engineering. Recently, a research group demonstrated the effect graphene doping in P3HT to realize good contact between the interface of P3HT and carbon.^246^ Because of the high conductivity and surface area of graphene, it was found that the P3HT/graphene composite developed superior contact with carbon, and hence depicted an enhanced PCE of 17.5% with the *J*_sc_ of 22.3 mA cm^−2^, while the device with only P3HT as the HTM showed a PCE of 11.1% with the *J*_sc_ of 19.3 mA cm^−2^. Besides conducting polymeric materials, several researchers also depicted the potential of small molecule-based HTMs. Small molecules such as copper phthalocyanine (CuPc), triazatruxene (TAT), 5,10,15-triphenyl-5*H*-diindolo[3,2-*a*:30,20*c*]-carbazole (TPDI), and BDT2MeDPA (unit composed of difluorobenzene, benzo[1,2-*b*:4,5-*b*′]dithiophene (BDT) and 4,4′-dimethoxydiphenylamine (DPA) groups) also depicted their utility for the enhancement of the device parameters in carbon-based PSCs.^248,253–258^ Habisreutinger *et al.* reported that the use of an HTL material composed of CNT/polymer in PSCs prevented thermal degradation. This group reported a high efficiency of 15.3% with an average efficiency of 10% ± 2%.^259^ Yoon *et al.* reported the preparation of an HTL layer of CNT/PEDOT:PSS for PSCs to improve the properties of PEDOT:PSS, and subsequently to decrease the hole vacancies in the PSCs. Because of the improvement in the HTL, this group reported a high PCE of 16% with almost zero hysteresis.^260^ Batmunkh *et al.* demonstrated SWCNTs/TiO_2_-based nanofibers to increase the rate of electron transportation.^261^ However, during the fabrication and characterization of PSCs, the hysteresis effect, losses due to recombination and carrier extraction are some of the major challenges faced by various researchers, which must be overcome to realize a perfect device. This challenge was somewhat addressed by doping graphene quantum dots (GQDs) in the thin film of tin oxide (SnO_2_), which not only improved the PCE (20.23%), but also reduced the hysteresis effect.^262^ Further, Zhu *et al.* incorporated graphene to improve the properties of SnO_2_ as an ETL to enhance the electron mobility and decelerate the charge recombination process in PSCs. This group reported an improvement in efficiency from 15.45% to 17.01% and 17.36% to 18.11% in the forward and reverse scan, respectively.^263^ Additionally, carbon-based nanomaterials can also be used in the perovskite layer to enhance the electric properties of PSC devices. It has been reported that the introduction of graphene-based materials in the perovskite layer can efficiently improve the short circuit current and fill factor of PSCs by improving the surface area and lower resistance.^264^ Briefly, the HTMs play a significant role in enhancing the device parameters in carbon-based PSCs. However, strong contact must be established between the HTM and carbon electrode to produce a highly efficient device.

## 6. Electron transport materials in carbon-based PSCs

**Fig. 8:**
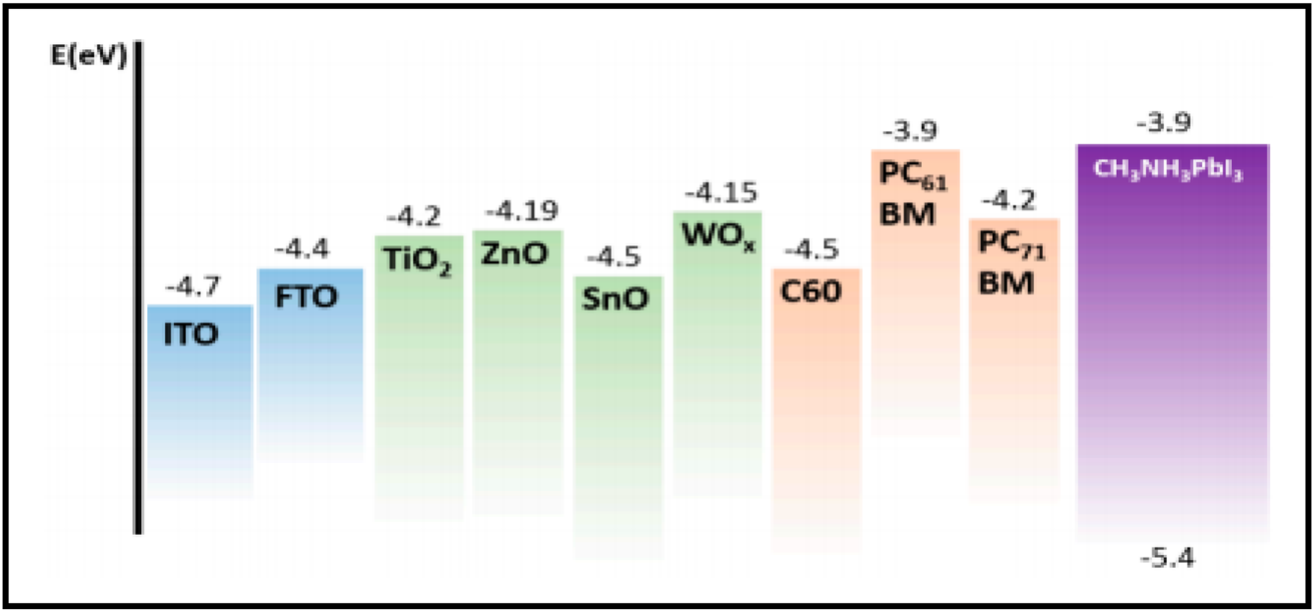
Energy levels of different ETMs and perovskite [reproduced from ref. 272 with permission from American MDPI, Copyright 2020].

The electron transport materials (ETMs) play a very significant role in PSCs to extract electrons from the excitons generated in the perovskite layer, and thus simultaneously reduced the recombination rate of electron and holes between the TCE and perovskite layer. An ETL with minimum pin holes and high electrical conductivity is regarded as the best ETL for PSCs. It has been reported that an effective ETL not only enhances the PCE of the devices, but also reduces their hysteresis behaviour, resulting in accurate PCE measurements. Also, good interfacial contact between the ETL and perovskite layer decides the overall performance of PSCs. Generally, in a good device, the series resistance (*R*_s_) value should be low, while the shunt resistance (*R*_sh_) and recombination resistance (*R*_rec_) values must be high. Hence, the energy level of the ETL must be well matched with the active layer for the smoother facilitation of charge transportation.^265^ TiO_2_ is a widely accepted ETM in DSSCs and PSCs.^267^ It possesses a good conduction band of −4.4 eV, with a valence band maximum of −7.63 V. Because of these appropriate values, TiO_2_ usually shows good properties for electron transportation;^268^ however, it generally requires a high-temperature sintering process. In this case, several methods have been reported for the fabrication of TiO_2_ at low temperature. For example, the colloidal spray coating method, which allows the coating of TiO_2_ up to 100 °C.^269^ The performance of TiO_2_ depends on several factors, which must be optimized to obtain good device parameters. Recently, it was demonstrated that an optimized thickness of the TiO_2_ layer can significantly minimize the charge recombination in the devices, thus enhancing the PCEs.^270^ Further, it was also found that the surface treatment of the TiO_2_ layer greatly affects the device parameters. To demonstrate this behaviour, a group of researchers used titanium tetra chloride (TiCl_4_) to cover the surface of TiO_2_ and reported improved device parameters.^271^ However, because of the high rate of recombination in TiO_2_, other ETMs such as ZnO, SnO, WO_*x*_, C_60_, PC_61_BM, and PC_71_BM have also been investigated in the last few years. Fig. 8 shows the energy level diagram of these ETMs together with the perovskite material.^272^ Among them, ZnO (−4.19 eV) showed excellent behaviour as an ETM in PSCs due to its high electron conductivity, low sintering temperature and solution processability. Additionally, ZnO has a direct band gap 3.3 eV, enabling it to behave as a good ETL in PSCs. However, because of its high chemical instability, ZnO degrades rapidly, which reduces the stability of the devices.^273,274^ Recently, the focus has shifted to SnO_2_ as a promising and efficient ETL for PSCs. SnO_2_ can be prepared by both low-temperature and high temperature processes; however, low temperature-processed SnO_2_ shows better electrical (∼10^−3^ cm^−1^ V^−1^ s^−1^) and optical properties.^275,276^

**Fig. 9:**
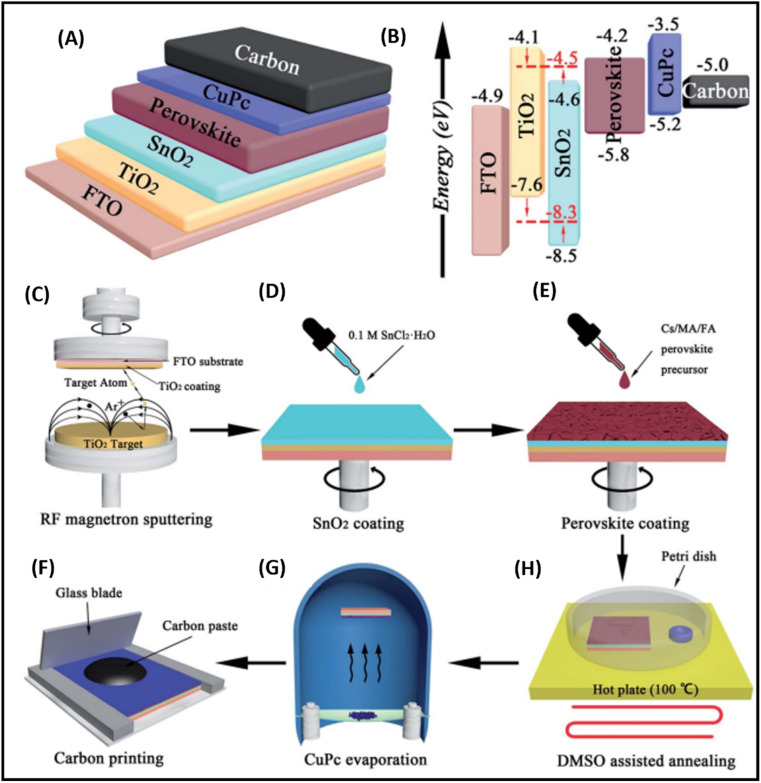
(A) Device structure of the TiO_2_/SnO_2_ ETL-based carbon-PSC, (B) energy level diagram corresponding to the device structure, and (C–H) fabrication process for the commissioning of PSCs [reproduced from ref. 283 with permission from The Royal Society of Chemistry, Copyright 2018].

Furthermore, SnO_2_ shows anti-reflection properties with a negative ultraviolet (UV) photocatalysis effect, making it a good candidate as an ETM. The first SnO_2_-based PSC was depicted by Tingli Ma *et al.* in 2015 with a PCE of 6.87%.^277^ Subsequently, several groups reported the preparation of SnO_2_-based ETLs for PSCs.^278–281^ In 2016, Grätzel *et al.* reported a breakthrough by demonstrating a PCE of nearly 21% in SnO_2_-based ETLs for PSCs, in which the fabrication of the SnO_2_-based ETL was carried out *via* a low-temperature chemical bath technique.^282^ However, the previously reported work on SnO_2_-based ETLs showed the problem of pinholes, which must be overcome to prevent a leakage current and charge recombination at the interface of the ETL and perovskite. Hence, it was suggested that bilayer ETLs can significantly overcome these obstacles. In this regard, Liu *et al.* demonstrated a TiO_2_/SnO_2_ bilayer ETL for carbon-based planer heterojunction PSCs. In this work, it was depicted that the TiO_2_/SnO_2_ bilayer ETL not only improved the electron extraction ability, but also reduced the rate of charge recombination. The TiO_2_ layer was fabricated *via* the radio frequency magnetron sputtering technique, while the SnO_2_ layer was fabricated by the double spin-coating of 0.1 M ethanolic solution of SnCl_2_·2H_2_O at 5000 rpm for 30 s, and subsequently annealing for 1 h at the temperature of 195 °C (Fig. 9). After the fabrication of the complete device, it was shown that the champion device depicted a *V*_oc_ of 0.98 V, *J*_sc_ of 23.28 mA cm^−2^, FF of 67%, and finally an appreciable PCE of 15.39%. Again, a high stability of 1200 h was shown for this champion device.^283^ In addition to inorganic ETMs, organic electron transport layers also depicted promising results. Among the carbon-based ETMs, fullerene-based derivatives have mainly been employed as electron transport materials. Although PCBM-based materials show sufficient conductivity and charge carrier mobility, only few works have been reported on PCBM-based PSCs. Due to the use of the same type of solvent system (DMSO and DMF) for both PCBM and perovskite, it becomes difficult to control the uniformity of both layers, and consequently low PCEs are obtained for PCBM-based PSCs.^284^ Hence, modified crosslinking fullerene derivatives were also synthesized, which showed solvent resistance towards DMF-like solvents.^285^

## 7. Stability and sustainability of carbon-based PSCs

The stability and sustainability of carbon-based PSCs have attracted great interest from researchers, thus becoming a research focus. Meng *et al.* reported the fabrication of an HTM-free carbon-based PSC with C_60_ as the electron transport material (ETM). Subsequently, the fabricated device was tested under 1 sun illumination at the maximum power point (MPP). It was found that the devices fabricated without encapsulation retained almost 95% of their initial PCE in air at the RH of 40–60% after 180 h.^286^ Zhou *et al.* demonstrated all-air processed PSCs using air-stable MWCNT-incorporated FA_*x*_MA_1−*x*_PbI_*y*_Br_3−*y*_ perovskite films.^287^ Here, the use of MWCNT was done to mediate the crystallization process of the perovskite phase and to prevent the moisture effect. The champion device exhibited a PCE of 16.25%, while the air stability for 500 h of the fabricated devices at the MPP with RH of 30–80% was found to be 94.9% of the initial PCE. Alternatively, the pristine devices only retained 23.4% of their initial PCE after 200 h. Bashir *et al.* demonstrated the utility of copper-doped nickel oxide (Cu:NiO_*x*_) for minimizing the recombination resistance and improving the photocurrent.^288^ The fabricated device showed high stability for 60 h with 100% maintenance of its initial PCE. Further, this group also fabricated a monolithic perovskite module with an active area of 70 cm^2^ over the glass substrate (100 cm^2^) and reported a significant PCE of 12.1% for the champion module. Zhou *et al.* reported the fabrication of TiO_2_-free carbon-based PSCs and used solution-processable hexamethonium bromide (HMB)-doped C_60_ as the alternative to TiO_2_.^289^ The fabricated device with HMB-doped C_60_ exhibited remarkable stability for 338 h at 1 sun condition by maintaining 90% of its initial PCE at the MPP. Some researchers investigated how the device parameters of PSCs can be enhanced. For example, Kapoor *et al.* showed the effect of excess PbI_2_ in PSCs.^290^ The fabricated device was analyzed for 68 h at 25 °C and 65% RH. The ageing test showed that the standard device maintained its current, whereas the device containing 15% excess PbI_2_ showed a reduction in current by 10%. Aitola *et al.* incorporated low-cost single-walled carbon nanotubes (SWCNTs) as the hole contact material and reported long-term stability for SWCNT-based PSCs.^239^ At MPP, 1 sun illumination, N_2_ atmosphere and at 60 °C, the stability of the SWCNT-based devices was compared with that of the standard devices. It was found that standard devices with Au as the back electrode and spiro-OMeTAD as the HTM showed a drastic change in PCE in the period of 140 h, while the SWCNT-contacted devices demonstrated a very small change in PCE with a good lifetime of 580 h. Raminafshar *et al.* showed that the hydrophobic nature of carbon-based electrodes effectively protects the PSC devices from moisture, and thus improves their stability. This group reported that the PCE of their carbon-based PSCs did not change even after four months of their fabrication without any encapsulation. These devices were kept at the ambient temperature of 25 °C and humidity level of 20% to 30%.^291^ Jiang *et al.* reported the preparation of a conducting film of carbon and graphite with a relative sheet resistance of 4 ohm per sq.,^292^ while Chu *et al.* reported the preparation of a conducting electrode composed of carbon and p-type NiO nanoparticles, achieving a promising PCE of 13.26%.^293^ In addition to the improve efficiency using carbon-based electrodes, stability-related studies have also been done, revealing that carbon-based electrodes not only improve the PCE but also the stability of the PSCs. Zhang *et al.* reported the preparation of a self-adhesive carbon film with microporous morphology as an electrode system for PSCs and reported a high efficiency of 19.2% and showed that the fabricated device retained more than 94% of its initial PCE after 80 h, while the PSC device prepared *via* the conventional method exhibited a PCE of 15.2% and possessed lower stability than the carbon-based PSCs.^294,295^ Siram *et al.* reported a comparative study of organic nanocrystal-modified MWCNTs and conventional electrodes of Au for PSCs and showed that the PSCs with the organic nanocrystal-modified MWCNTs depicted very stable behaviour after 60 days, while the PSCs with the conventional electrodes of Au degraded rapidly and lost their efficiency.^296^ All these works showed the extensive use of carbon-based materials for the enhancement of stability among PSCs. The literature database of the reported carbon-based PSCs mainly focused on the role of carbon in the enhancement of the stability and durability; however, the self-stability of the perovskite is still not clearly understood. The chemical inertness of the perovskite layer must be explored to determine the actual stability of PSCs. However, the present status about the stability gives direct evidence that carbon-based materials are the only successful candidates for enhancing the lifetime of PSCs. The hydrophobic properties of carbon-based materials have been proven as a shield to protect from moisture. Further, these materials are not involved in the formation of halides, as found in the case of metal-based PSCs.

## 8. Manufacturing and processing of large-area modules

**Fig. 10:**
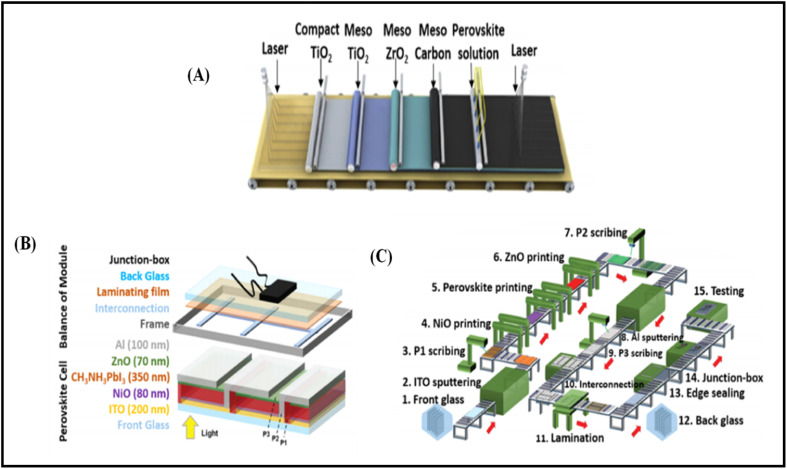
(A) Schematic of the process for the fabrication of carbon-based mesoscopic PSC modules [reproduced from ref. 303 with permission from John Wiley & Sons, Copyright 2017]. (B) Schematic of the integrated monolithic PSC modules. (C) Manufacturing line for the fabrication of monolithic PSC modules [reproduced from ref. 304 with permission from The Royal Society of Chemistry, Copyright 2017].

Although several efforts have been devoted to the laboratory-scale development of PSCs modules, the fabrication of large-scale modules is expected due to the scalability of carbon-based PSCs. Specifically, the solution processability of carbon-based materials opens a new door for the batch-scale production of carbon-based modules.^297,298^ However, although some manufacturing routes have been explored, simple and cost-effective manufacturing routes need to be developed. Because of the low dispersibility of carbon-based materials, their solution processability makes the fabrication of substrates with these materials challenging. In this case, CVD-like techniques for the fabrication of graphene- and CNT-based electrode systems somewhat alleviates the dispersion issues, but the scalability of these techniques is limited.^299–302^ Hence, researchers are focusing on alternative ways to disperse carbon-based materials. In this regard, the development of carbon-based inks and paste has been reported, which can be used in a variety of printers. The inkjet printing technique has been depicted as a cost-effective printing technique, which can easily implemented for large-scale module preparation. However, the ink that is implemented for inkjet printing must possess a low boiling point, non-polar nature and be inert to the perovskite materials. Another aspect that must be understood before producing large-scale modules is the series of layers and their corresponding thickness in the modules. Fig. 10(A) shows the proposed production line of a carbon-based PSC panel of 1 m^2^, where three mesoporous layers of TiO_2_, ZrO_2_ and carbon are printed on the top of the compact layer of TiO_2_, respectively. Subsequently, perovskite solutions are drop-casted over the scaffold of these three mesoporous layers.^303^ Further, each panel consists of about 96 perovskite solar modules, where each module constitutes different quantity of subcells. Further, a group of researchers also depicted a proposed schematic for the modules of monolithic PSCs, where a laser-based system was shown to separate the layers and subcells (Fig. 10(B)). Interestingly, this group also depicted a full fabrication line for the commissioning of panels of PSCs (Fig. 10(C)), where P1, P2, P3 in the fabrication line depict the laser scribing process for the separation of the individual layers and subcells, respectively.^304^ However, although various schematics have been proposed for the manufacturing of large-scale PSCs, there are still gaps in the degradation study and stability of the panels under harsh conditions, which must be addressed to realize the complete availability of PSCs in daily applications.

## 9. Economic viability and cost-benefit analysis

Presently, despite the low PCEs and stability of PSCs in comparison to silicon-based solar cells, PSCs show a relatively lower fabrication cost than silicon-based solar cells. The rapid development of the synthesis methods for carbon-based materials *via* cost effective techniques shows that the cost for the fabrication of carbon-based materials can be reduced significantly.^36^ Song *et al.* proposed and depicted the best route for their fabrication with a cost-benefit analysis. Accordingly, the per m^2^ average cost of a PSC module should be $31.7, with the minimum sustainable price of $0.41 per peak DC watt. This cost covers the fabrication of each component including PSC commissioning, integration of the modules, sealing, and finally performance testing. Comparatively, carbon-based materials show greater economic viability and sustainability in terms of the per watt cost than the silicon-based crystalline solar cells, where their per watt cost is around $0.4 to $0.5.^304^

## 10. Challenges and possible solutions

**Fig. 11:**
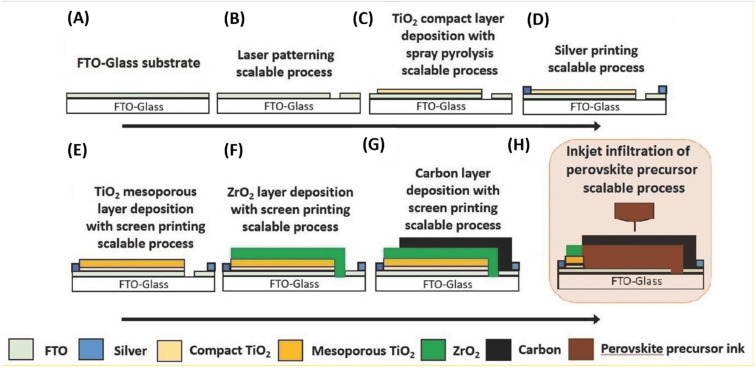
Pictorial representation of the fabrication of a complete carbon-based PSC. (A–H) Different stages of the fabrication in a scalable process [reproduced from ref. 305 with permission from John Wiley & Sons, Copyright 2016].

Carbon-based PSCs have reached a rapid development stage in recent years, although with the evolution of these types of PSCs, several challenges have been identified by researchers. The exploration of the identified problems revealed various facts about carbon-based PSCs. A simple fabrication process requires the drop-casting of the perovskite precursor over the carbon-based substrate, where, generally, it is assumed that the perovskite precursors infiltrate the carbon layer and develop into a perfect perovskite phase. Fig. 11 shows a pictorial representation of the fabrication of mesoporous carbon-based PSCs, which depicts the various stages in the fabrication of each component to make a complete device.^305^ However, due to the different types of infiltration mechanisms, it becomes difficult to infiltrate the perovskite precursor completely. Hence, the first challenge is to develop and optimize a cost-effective infiltration technique to achieve precision in the devices. Among the different types of infiltration techniques, the inkjet printing technique is regarded as the fastest technique for the large-scale fabrication of PSCs with remarkable reproducibility and batch-level productivity.^305^

**Fig. 12:**
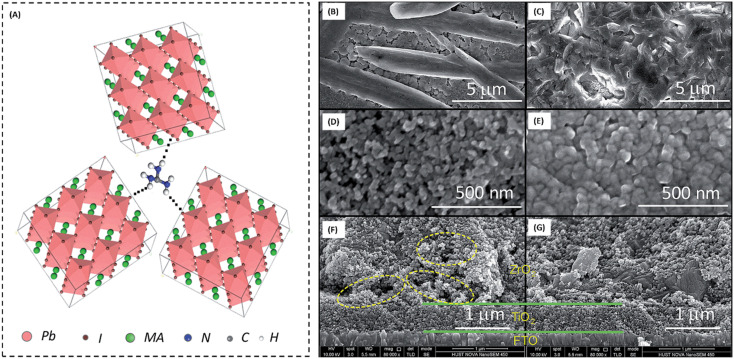
(A) Cross-linked perovskite grains under the effect of GuCl, (B and C) SEM images of MAPbI_3_ and MAPbI_3_ + *x*GuCl on FTO glass substrates, (D and E) SEM images of MAPbI_3_ and MAPbI_3_ + *x*GuCl infiltrated in m-TiO_2_ layer, and (F and G) SEM cross-sectional images of m-TiO_2_ layer and m-ZrO_2_ with MAPbI_3_ + *x*GuCl (*x* = 0.25) perovskite [reproduced from ref. 313 with permission from The Royal Society of Chemistry, Copyright 2017].

However, the quick conversion of the perovskite liquid precursors into perovskite crystals blocks the nozzles of the inkjet printers, and consequently it becomes difficult to continue the process for longer times. Further, the same problem also hinders the use of other materials, thus showing one of the challenges faced by the researchers. To resolve this issue, either new inkjet printing techniques or new deposition techniques should be developed, or new types of precursors must be explored, which show a slow conversion rate of crystallization. Hence, it is another issue to slow down the rate of crystallization by maintaining the infiltration flow rate. A solution to this problem may be controlling the crystal size of the perovskite. The use of additives or dopants can significantly control the perovskite crystal growth by reducing the rate of crystallization in the mesoporous layers of the device. Additives such as 5-ammoniumvaleric acid iodide (5-AVAI) have demonstrated promising behavior for the proper and balanced growth of the perovskite in the cavities of the TiO_2_ layer.^306^ Other additives such as [6,6]-phenyl-C_61_-butyric acid methyl ester (PCBM), carbon quantum dots (CQDs), benzylamine hydroiodide (BA), liquid metals and 4-(aminomethyl) benzoic acid hydroiodide (AB) also showed potential to enhance the device parameters of carbon-based PSCs.^307–310^ Guan *et al.* reported the use of PCBM as an additive in perovskite precursor solutions to improve the device parameters by modifying the surface morphology of the perovskite.^311^ They mixed PCBM with two types of perovskite precursors, namely, MAPbI_3_ and MAPbI_2.95_ (BF_4_)_0.05_, and found that the PCEs of the devices improved from 8.58% to 12.36% for MAPbI_3_ + PCBM (0.25 mg mL^−1^) and 12.77% to 14.26% for MAPbI_2.95_ (BF_4_)_0.05_ + PCBM (0.25 mg mL^−1^), respectively. This group reported that the incorporation of PCBM not only improved the surface morphology of the perovskite, but it also reduced the fast process of charge recombination, and thus resulted in improved device parameters. Continuing the work related to MAPbI_(3−*x*)_(BF_4_)_*x*_, Chen *et al.* depicted that upon the replacement of iodide ions with BF_4_^−^, both the conductivity and light-harvesting ability were enhanced.^312^ Metallic doping in the perovskite precursor was demonstrated by Hou *et al.*, who demonstrated the use of guanidinium chloride (GuCl) as an additive in the perovskite precursors to enhance the PCE of the device.^313^ It was reported that the cation of GuCl helped to cross-link the adjoining perovskite units, and thus provided a defect free morphology in the perovskite crystals (Fig. 12).

The *V*_oc_ of the GuCl-doped perovskite-based device was found to be 1.02 V, while the device with only a perovskite layer depicted a *V*_oc_ of 0.88 V. Similarly, Sheng *et al.* depicted the doping of Li ions in perovskite by incorporating LiCl in the perovskite precursor.^314^ It was depicted that the Li-doped perovskite showed faster electron transfer, and thereby the addition of 30 wt% of LiCl to the perovskite precursor resulted in a good PCE of 14.5% in the carbon-based PSCs. Recently, Zong *et al.* depicted the use of ammonium chloride (NH_4_Cl) in the precursor solution and depicted that the introduction of the ammonium ion in the framework of the perovskite creates a synergistic effect with the perovskite grains, and hence enhances the stability of the PSCs.^315^ Presently, different types of additives have been reported, but only a few showed viability for commercial exploitation. Table 3 summarizes some of the additives used recently in perovskite precursors for carbon-based PSCs.

**Table 3:** Different types of additives used in perovskite precursors

S. no.	TiO_2_	ZrO_2_	Carbon (μm)	Additive/s	PCE (%)	Ref.
1	0.5 μm	1.4 μm	10–12 μm	5-(AVA)_*x*_	10.46 for 31 cm^2^ and 10.74 for 70 cm^2^	316
2	1 μm	2 μm	10 μm	5-(AVA)_*x*_	10.4 cm^2^	317
3	1 μm	2 μm	10 μm	(AVA)_*x*_	11.2	318
4	0.5 μm	1.3 μm	10–12 μm	5-(AVA)_*x*_	11.06	319
5	600 nm	2 μm	12 μm	PCBM (0.25 mg mL^−1^)	12.36	311
6	600 nm	2 μm	12 μm	MAPbI_2.95_(BF_4_)_0.05_	12.77	311
7	600 nm	2 μm	12 μm	MAPbI_2.95_(BF_4_)_0.05_ + PCBM (0.25 mg mL^−1^)	14.26	311
8	_	_	_	MAPbI_3_ + 5% CQDs	7	311
9	500 nm	2 μm	15 μm	MAPbI_3_ + 5-aminovaleric acid hydroiodide (AVA)	14.1	309
10	500 nm	2 μm	15 μm	MAPbI_3_ + benzylamine hydroiodide (BA)	12	309
11	500 nm	2 μm	15 μm	MAPbI_3_ + 4-(aminomethyl) benzoic acid hydroiodide (AB)	15.6	309
12	1 μm	2 μm	10 μm	MAPbI_3_ + *x*GuCl	14.35	15

Further, the proper growth of the perovskite crystals in the mesoporous layers of TiO_2_ and ZrO_2_ also depends up the physical properties of the liquid perovskite precursor and the surface topology of the mesoporous layers. It is highly desirable that the liquid perovskite precursor should have an optimized viscosity to enable the control of the flow rate. Further, the droplet size of the perovskite precursor ink and its releasing time also affect the device parameters. Additionally, the mesoporous layers should have an appropriate pore size and surface, which smoothly allow the passage of the perovskite precursors.^316,320,321^ The contact of the perovskite crystals with the particles of TiO_2_ must be improved to obtain good device parameters. Thus, the size of the TiO_2_ nanoparticles must be maintained to ensure good contact with the perovskite crystals. Additionally, the annealing temperature must be maintained after the infiltration of the perovskite precursors. Yang *et al.* reported the size selectivity study for m-TiO_2_ particles and found that the device with the TiO_2_ particle size of 25 nm depicted the highest efficiency of 13.4%, while the devices with 10, 15, 20 nm TiO_2_ showed comparatively lower PCEs.^322^ Alternatively, Liu *et al.* reported that the optimized layer thickness of m-ZrO_2_ should be 1 μm.^323^

It has been reported that different types of solvent systems also affect the device parameters. Hence, the choice of solvent also affects the performance of the devices. This study was supported by Chen *et al.*,^324^ who investigated four polar solvents, *i.e.*, DMSO, DMF, γ-butyrolactone (GBL) and 1-methyl-2-pyrrolidinone (NMP). The phase study and device performance analysis suggested that DMF and DMSO possessed better wettability, and thereby resulted in good device parameters. The champion device with a fixed proportion of DMF and DMSO showed a good PCE of 13.89%. Thus, this work proved that the wettability of the perovskite precursor solution greatly affects the device performance, which is again dependent on the choice of solvent system.

The third challenge is the establishment of better contact between the perovskite phase and carbon electrode, which must be improved to achieved good device parameters. Additionally, the contact between TiO_2_ and perovskite phase should be uniformly established to realize the optimum device parameters. Liu *et al.* improved the contact between TiO_2_ and the perovskite interface by introducing an intermediate layer of silane.^325^ This silane layer was prepared by immersing mPSCs in an isopropanolic solution of aminopropyltrimethoxysilane (C_6_H_17_NO_3_Si /0.05 mM) for a few hours. It was shown that this process resulted in strong contact between titanium and silicon by developing Si–Ti bonds and suppressed the charge recombination process, and thus improved the PCE of the devices. The champion device with silane as the interfacial layer between TiO_2_ and the perovskite layer depicted a PCE of 12.7%.

Besides the above-mentioned challenges, the stability issue is one of the main challenges. Presently, there is huge gap among the systematic studies on the stability, charge transportation properties and degradation studies of the perovskite layer in carbon-based PSCs. Although several studies have been performed and showed high stability of up to 10 000 h in a protected environment,^318^ the stability of carbon-based PSCs under harsh environmental conditions needs more attention. Additionally, the search for efficient materials/dopants is necessary to get maximum information on the stability of carbon-based PSCs. In this case, guanidinium (Gu)-based materials have been reported as suitable stability enhancers.^326^ Similarly, cesium-based materials have also been reported as both efficiency and stability enhancers among the family of PSCs.^327^ Further, it has been reported that the addition of a triple cation and quadruple-cation to the matrix of the perovskite precursor enhances the efficiency and stability of metal-based PSCs. However, this type of work has not been reported for carbon-based PSCs due to the limited infiltration efficiency of the mesoporous layers. However, the research progress in overcoming these challenges shows that carbon-based PSCs will soon be introduced for commercial exploitation.

## 11. Future prospects and conclusions

Carbon-based materials have showed significant development in the last few decades. Because of their high conductivity, vast surface area and excellent optoelectronic properties, many carbon-based materials have shown potential as electrode materials in PSCs. The present review summarized the most prominent and advance works regarding the development of carbon-based PSCs. The procedures for the fabrication of carbon-based electrodes and scale-up methods such as screen-printing, slot-die coating, ink-jet printing and electro-deposition methods for carbon-based PSCs were clearly depicted. Graphite, carbon black, carbon nanoparticles, graphene and carbon nanotubes have been demonstrated as potential candidates for the fabrication of the back electrodes in PSCs, while graphene and CNTs also showed potential as material for the front TCEs in PSCs. Additionally, this review focused on the cost-effective methods for the preparation of graphene and CNTs *via* different methods. These carbon-based materials were successfully demonstrated as potential electrode materials for the enhancing the lifetime of PSCs. However, to date, all the reported carbon-based PSCs are lagging behind Au-based PSCs in terms of the PCEs, *J*_sc_ and FF values. The poor contact between the carbon-based electrode/perovskite interface and high resistance of the carbon electrodes are major reasons for the poor PCEs of carbon-based PSCs. Hence, solutions are urgently demanded to meet the huge marketing demands. The present review also summarized the possible solutions for the obstacles associated with carbon-based PSCs. Interface engineering between carbon and the perovskite phase can enhance the device parameters, while the incorporation of conducting nanoparticles in the carbon matrix can reduce the sheet resistance of carbon electrodes. Further, to develop cost-effective carbon-based back electrode systems in PSCs, cost-effective carbon materials are also required, which depends on the process for their synthesis. In this case, the use of solid waste-derived carbon materials can become a cost-effective method for the large-scale production of carbon materials as the back and front electrodes in PSCs. However, there are no investigations on the use of carbon materials derived from solid waste materials in PSCs. Also, the stability and sustainability of carbon-based PSCs still need to be reorganized according to the market demands. Although carbon-based PSCs demonstrate several advantages in terms of stability than metal electrode-based devices, the individual stability of each component in carbon-based devices still needs to be investigated for building a sustainable industrial symbiosis process.

## Author contributions

Conceptualization, S. P.; data curation, S. P., M. K.; formal analysis, G. T. and L. P.; funding acquisition, M. J. L., and N. G. S.; investigation, D. B. and S. P.; methodology, S. P.; resources, M. J. L., and N. G. S.; software, S. P.; supervision, M. J. L., and N. G. S.; validation, S. P., and R. S.; visualization, M. J. L., and N. G. S.; writing—original draft, S. P.; writing—review and editing, S. P., M. K., R. S, and L. P. All authors have read and agreed to the published version of the manuscript.

## Conflicts of interest

Authors declared no conflict of interest.

## Supplementary Material
